# Effect of Polymer Host on Aggregation-Induced Enhanced Emission of Fluorescent Optical Brighteners

**DOI:** 10.1021/acsapm.4c00090

**Published:** 2024-01-31

**Authors:** Zoé O. G. Schyns, Thomas M. Bennett, Gemma E. Davison, Michael P. Shaver

**Affiliations:** † Department of Materials, School of Natural Sciences, University of Manchester, Manchester M13 9BL, United Kingdom; ‡ Sustainable Materials Innovation Hub, Henry Royce Institute, University of Manchester, Manchester M13 9BL, United Kingdom; § ReCon^2^ Limited, Henry Royce Institute, University of Manchester, Manchester M13 9BL, United Kingdom

**Keywords:** aggregation-induced enhanced emission, solubility, dyes, fluorescence, smart materials

## Abstract

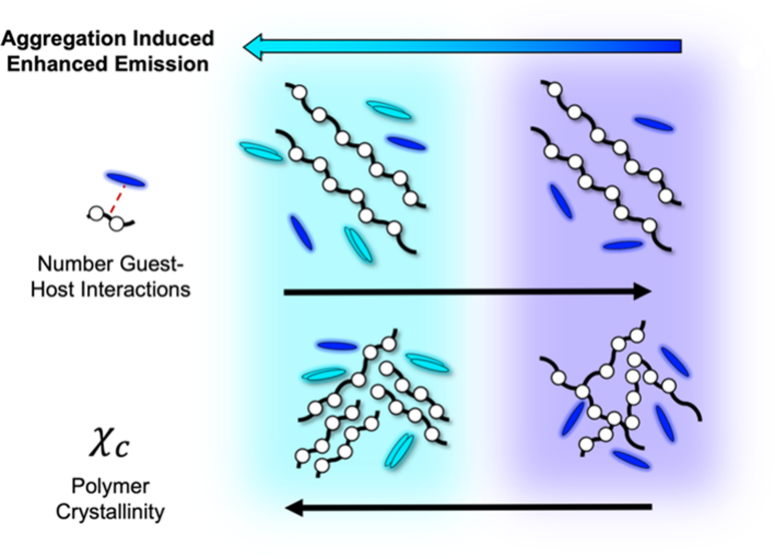

Fluorophores displaying concentration-dependent luminescence are becoming increasingly valuable in stress-sensing, tagging, and dyeing applications, including the quantification of recycled content in plastic packaging. In this work, we investigate the effects of the polymer matrix, dye structure, and crystallinity on aggregation-induced enhanced emission (AIEE). We demonstrate that the aggregation threshold required for successful quantification can be adjusted through modulation of guest–host (dye–polymer) interactions and monitored using an array of fluorescence characterization. Modification of guest–host interactions is realized through choice of host, change of guest, and tuning of the crystallinity of the host system. Increasing the number of guest–host interactions and solubility between guest and host, loosely predicted through the calculation of the solubility parameter, increases the aggregation threshold relative to other low-polarity and low-interacting systems. We demonstrate that issues, such as loading level and cost, associated with high aggregation thresholds, can be circumvented by increasing system crystallinity, improving spectral intensities, and subsequent quantification. These insights explore the fundamental understanding of supramolecular interactions that govern dye–polymer systems.

## 1 Introduction

Aggregation-induced enhanced emission (AIEE) or quenching (AIQ) is a concentration-based phenomenon that describes changes in emitted light by fluorophores. This behavior can be exploited for use in quantitative stress-sensing, biological sensors, and more. We previously reported on exploiting the aggregachromic behavior of 4,4-bis(2-benzoxazolyl) stilbene (BBS) to mark recycled content in poly(ethylene terephthalate) (PET), high-density poly(ethylene) (HDPE), and polypropylene (PP) packaging plastics.^1^ Quantitative analysis of fluorescence emission, lifetime measurements, and complementary digital color analysis yielded linear relationships between these fluorescence parameters and increasing recycled content. BBS′ AIEE behavior has also been demonstrated to allow accurate stress sensing in thermoplastics, by producing a linear fluorescence intensity increase with larger strain deformation.^2,3^

BBS belongs to a class of conjugated π-system fluorophores that display aggregation-induced enhanced emission (AIEE). This aggregachromic phenomenon occurs when fluorophores are dispersed throughout a continuous matrix—in most cases rigid—and form energetically favorable aggregated complexes at elevated concentrations.^4,5^ New intermolecular interactions arising between the aggregated molecules, such as H- or J-aggregates, modulate the electronic excited states of the individual molecules.^6^ Thus, the resulting aggregates can display distinct photophysical properties relative to their monomeric counterparts.^4,6^ BBS is an example of a J-aggregating fluorophore, whereby a negative Coulomb coupling between aggregated molecules reduces the first excited state energy, red-shifting and enhancing fluorescence emission.^6,7^

Quantitative analysis of AIEE phenomena, such as for stress-sensing and recycled content determination, requires low dye solubility and low aggregation thresholds within a given polymer matrix. Threshold aggregation concentrations in fluorescing and nonfluorescing dye–polymer (guest–host) systems are reported to depend on the crystallinity of the host matrix, the relative dye solubility, and the number of dye–polymer (guest–host) interactions.^8,9^ Dye–polymer composite systems generally have three different levels of interactions: (a) no interactions, (b) covalently linked, or (c) guest–host (dye–matrix) intermolecular.^9^ In systems with an increased number of guest–host interactions, the threshold concentration for aggregation is reduced as intermolecular interactions take precedence over dye–dye aggregation-inducing interactions.^9,10^ For example, the aggregation threshold of Disperse Red 1 (DR1) changes drastically when dissolved in matrices with increasing polarity: polystyrene (PS), poly(4-vinyl-phenol) (PVPh), or poly(styrene-sulfonic acid) (PSSA).^9^ An increased number of guest–host intermolecular interactions due to hydrogen bonds forming between the –OH present in PVPh and DR1, or the formation of an ionic complex between DR1 and the sulfonic acid group of PSSA, maximizes chromophore separation and results in an increased aggregation threshold.^9−12^

Dye molecules in dye–polymer composite materials routinely reside within mobile and permeable amorphous domains of the host matrices.^4,13^ By exploiting temperature-induced mobility within these systems, the fluorescence response can often be modulated to enhance or diminish aggregation effects.^3,13^ This temperature-dependent phenomenon has previously been reported in numerous dye–polymer systems, such as the appearance of excimer bands after annealing in 1,4-bis(*R*-cyano-4-octadecyloxystyryl)-2,5-dimethoxybenzene-PET blends.^3,14−19^

In AIEE-based research, the main objectives are often narrow, focusing on the fluorescence properties of a single class of molecules within singular polymer hosts. As processing conditions are a major factor in additive dispersion, we herein expand beyond typical approaches to comprehensively examine how dye structure, process temperature, percent crystallinity, and dye–matrix interactions affect AIEE behavior across a broad scope of common plastics. Leveraging extensive fluorescence characterization techniques and expansive guest and host scopes, we demonstrate that both the host and dye structure, specifically the level of aromaticity and chemical complexity of the polymer backbone or dye core, dictate the AIEE response of the system. Additionally, we show that AIEE response in poly(ethylene terephthalate) (PET) and poly(lactic acid) (PLA) can be modulated by changing the system’s crystallinity, improving the spectral resolution for use in sensing applications. These insights into the impact of polymer hosts’ properties on AIEE behavior can inform the development of selection criteria for molecular additives—whether synthetic or industrially sourced, in various domains ranging from sensing to dyeing. By highlighting the impacts of polymer host properties and dye structure on AIEE behavior, this work offers fundamental insights into guest–host systems for applications beyond recycled content determination.

## 2 Results and Discussion

### 2.1 Dye Structure Impact on Aggregation-Induced Emission

4,4-Bis(2-benzoxazolyl) stilbene (BBS), trade name Optical Brightener OB-1, while belonging to a class of AIEE exhibiting fluorophores, also belongs to a broad group of plastic additives known as optical brighteners (OBs) or fluorescent whitening agents (FWAs). OBs commonly have absorbance ranges within the UV region of the electromagnetic spectrum (<400 nm) and are characterized by emitting in the blue range of the visible spectrum (ca. 400–500 nm). These additives are often used to improve the appearance of plastic products. Incorporation of OBs lowers reflectance in the UV and near-visible and increases reflectance through fluorescence in the visible range, negating the yellow tint often seen in some recycled materials.^20^

**Figure 1:**
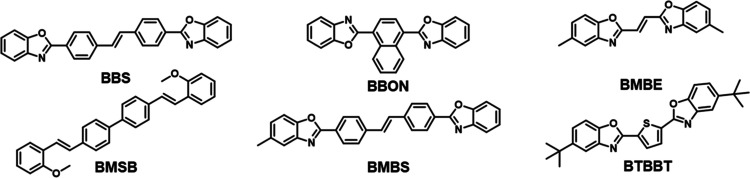
Screening AIEE behavior of commercially available optical brighteners in HDPE. Structures of (top row from left to right) BBS, BBON, and BMBE and (bottom row from left to right) BMSB, BMBS, and BTBBT.

**Figure 2:**
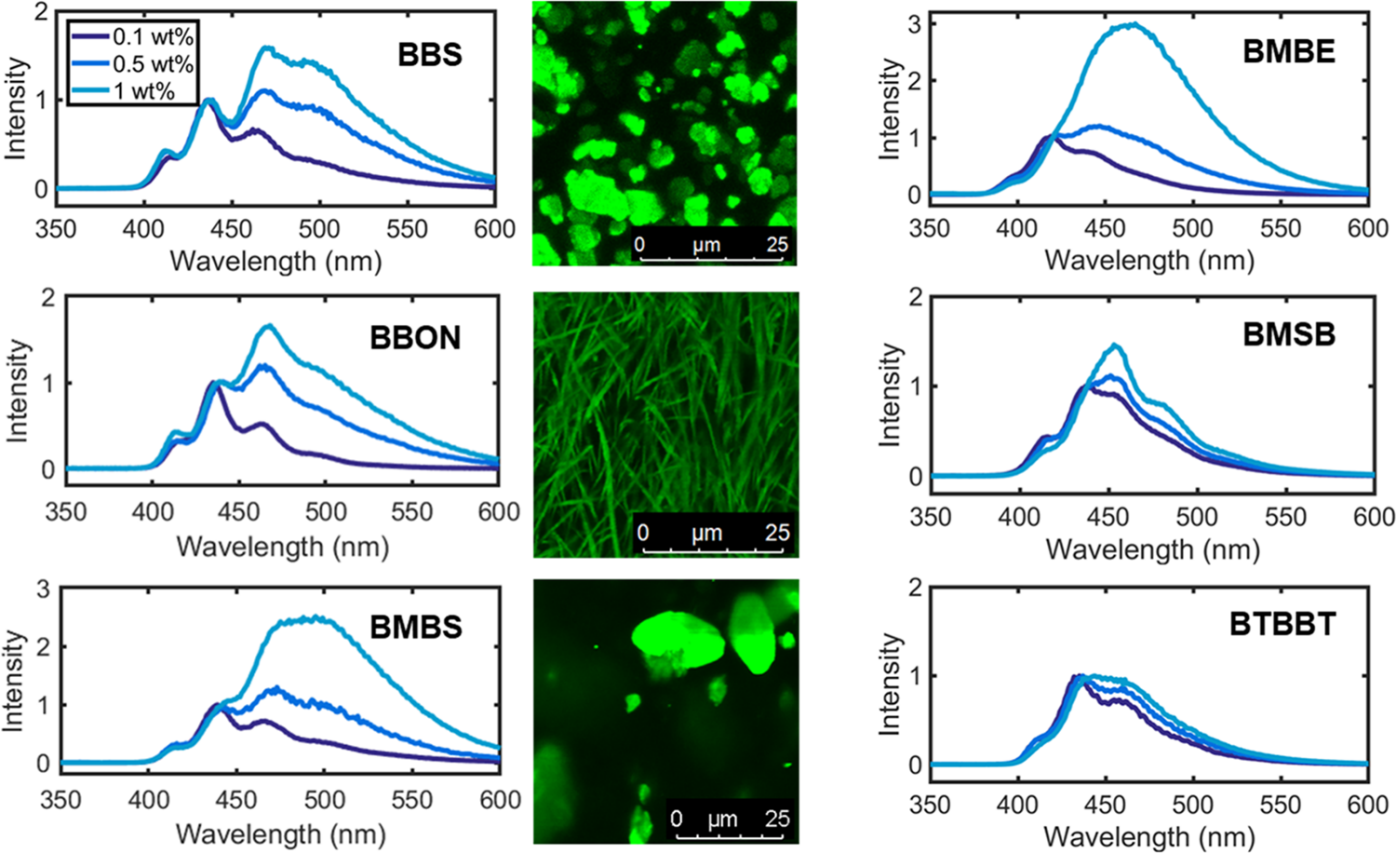
Screening AIEE behavior of commercially available optical brighteners in HDPE. Fluorescence emission spectra (0.1, 0.5, 1 wt % dye loading) and confocal microscopy images (0.5 wt % dye loading) of screened optical brighteners.

The effects of guest structure on aggregation-induced emission were investigated using loading studies of different fluorophores melt blended with high-density polyethylene (HDPE). Six commercially available optical brighteners with conjugated π-systems were chosen for initial screening: 1,4-bis(benzo[*d*]oxazol-2-yl)naphthalene (BBON), 4,4′-bis(2-benzoxazolyl) stilbene (BBS), 4,4-bis(2-methoxystyryl)biphenyl (BMSB), 2,5-bis[5-(*tert*-butyl)-1,3-benzoxazol-2-yl]thiophene (BTBBT), bis(5-methyl-2-benzoxazolyl)ethylene (BMBE), and 4-(2-benzoxazolyl)-4′-(5-methyl-2-benzoxazolyl)stilbene (BMBS) (Figure 1). The presence of aryl groups along the dye core (1) provides easily excitable electrons for fluorescence transitions and (2) limits the mobility of the dyes, preventing dispersion and promoting aggregation.^12^ Three concentrations (low—0.1 wt %, med—0.5 wt %, and high—1 wt % with respect to polymer matrix) of each optical brightener were melt blended with HDPE to test for AIEE behavior. All six OBs produced characteristic fluorescence emission spectra when irradiated in the UV range, with spectral features corresponding to two or more electronic transitions (Table 1 and Figure 2). The spectra were normalized to their respective fluorescence maxima to minimize the effect of sample dimensions, slit widths, and fluorimeter settings (Table 1 and Figure 2).

**Table 1:** Optical Brightner Fluorescence^a^

dye	monomeric peak (nm)	peaks (nm)	AIEE	ratio 1 gradient—∇	ratio 2 gradient—∇
BBS	415	436, 465, 500 (br)	Y	2.03^b^	2.42^b^
BMBS	439	413, 467, 500 (br)	Y	1.72	1.67
BMBE	418	440, 398, 470 (br)	Y	0.42	0.43
BBON	435	415, 460, 500 (br)	Y	0.41	0.35
BTBBT	435	458	N		
BMSB	437	413, 453, 480 (br)	N		

a Fluorescence characteristics of optical brighteners melt compounded with HDPE including monomeric peak taken as the fluorescence maxima, emission peaks, and presence of aggregation-induced enhanced emission. Gradients extracted from fits between ratios of AIEE peaks and monomeric peaks and increasing dye concentration (SI Figures S9–S11). Fitting was performed using the MATLAB curve fitting toolbox. Broad unstructured aggregated peaks are denoted as (br).

b Reference (1).

Of the 6 tested, dyes BBS, BMBS, BBON, and BMBE displayed evidence of aggregachromic behavior at the concentrations tested (0.1–1 wt % dye loading). These 4 dyes demonstrated red-shifted emission, which is typically associated with j-aggregate formation, as is the case for BBS.^21^ Further quantum calculations, beyond the scope of this manuscript, would be required to verify the exact aggregation mechanism of BBS, BMBS, BBON, and BMBE.^22^ Dyes BTBBT and BMSB displayed no evidence of AIEE at relevant concentrations (Figure 2). We hypothesize that BTBBT’s bulkier *tert*-butyl groups would pose a steric barrier to aggregation, preventing dye–dye stacking and increasing van der Waals interactions between dye and polymer, further reducing chance of aggregation.^23^ In the case of BMSB, the limited evidence of AIEE may be explained by the free rotation around the central biphenyl bond disrupting planarity and increasing the steric impact of ortho-methoxy groups. Due to this lack of AIEE, testing of BTBBT and BMSB was not conducted during larger-scale trials.

Fluorescence lifetime measurements are independent of fluorescence excitation wavelength and other experimental parameters, thus providing an accurate verification of fluorescence behavior.^24^ Corresponding fluorescence lifetime measurements were performed on samples showing AIEE (BBS, BMBS, BBON, and BMBE) at relevant concentrations (0.1–1 wt % relative to HDPE matrix). Fluorescence lifetime decays, recorded at the aggregation-enhanced emission wavelengths of the dyes (SI Figures S1–S8), were indicative of multiple contributing lifetime parameters; typically associated with multiple dye conformational states.^25^ Fitting multiexponential decay functions yielded two lifetime contributions corresponding to a short-lived (τ_1_) monomeric dye state and one long-lived (τ_2_) aggregated dye state (Fluorescence Lifetimes section). Increases in the τ_2_ contributions, stemming from increased presence of aggregate, were observed for increasing dye concentration for samples containing BMBS, BBON, BMBE, and BBS (SI Figures S1–S8). These increases were nonlinear due to the complexity of fluorescence responses of these systems at high dye concentrations [e.g., aggregation-induced quenching (ACQ), long-range ordering, nonlinearity, or charge transfer].^26−28^

Following successful identification of AIEE in four of the six chosen optical brighteners, scaled-up testing of BBON, BBS, BMBS, and BMBE was conducted using stress-sensing or other application-specific starting masterbatch (MB) concentrations of 0.1 wt %, in a process closely following previously reported work (Sample Preparation section).^1^ The MBs were diluted to fixed percentages (0.01–0.1 wt % BBS relative to polymer matrix). Fluorescence emission spectra measurements were performed on all of the diluted samples and spectra normalized to the fluorescence emission maxima of the chosen die (tabulated in Table 1 and SI Figures S9–S11). Fluorescence intensity ratios were calculated between aggregation-enhanced peaks (*I*_aggregate_) and those corresponding to the monomer (*I*_monomer_) according to eq 1 (summarized in Table 1).
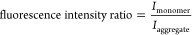
1Linear relationships were detected between the intensity ratio and dye concentration for all four of the OBs tested (Table 1, Figure 1 and SI Figures S9–S11). To estimate the relative AIEE strength of each system, a comparison can be made between gradients (∇) of the fit between intensity ratio and dye concentration (1st-degree polynomials—Table 1). Strong AIE behavior, or a low aggregation threshold, is characterized by a high gradient and vice versa for weak AIE behavior.

From these estimations, BBS displayed the strongest AIEE behavior in HDPE with increasing concentration (∇ = 2.42 {500.430 nm}),^1^ closely followed by its methyl-substituted analogue BMBS (∇ = 1.67 {500.430 nm}) (Table 1). This additional methyl group, situated on the dye’s peripheral substituted aryl ring, may sterically hinder stacking of the BMBS molecules, leading to a weaker AIEE response. This difference in the strength of AIEE with increasing concentration for the four dyes tested is attributed to structural inhibition of supramolecular interactions. From the experiments conducted, fluorophores featuring higher polarity and/or interactivity with the matrix (e.g., most apparent for BTBBT and BMSB) produce the lowest aggregachromic response. However, we hypothesize that the reasons for low AIEE observed for BBON and BMBE are more nuanced. BBON features a planar structure that can stack to create long-range complex charge transfer aggregate shapes, where ACQ is typically favored over emitted pathways. Meanwhile, BMBE lacks an aromatic core, and its fluorescence stems from its conjugated double bond which does improve luminescence (relative to BBON) but having less delocalized electrons could decrease fluorescence intensity overall relative to the other OBs tested (Table 1).^12^

Confocal microscopy was used to investigate the microscopic appearance and structural differences of the molecular aggregates within the HDPE matrix. By choosing an appropriate wavelength range corresponding to aggregate emission, aggregate-type structures were successfully detected in BBS-, BMBS-, BMBE-, and BBON-doped HDPE, with differing morphologies (Figure 1 and SI Figures S12–S15). Well-distributed spherical aggregates were detected in HDPE-BMBS samples as expected from fluorescence emission measurements (Figure 1 and SI Figure S12). In contrast, confocal images of BBON revealed fiber-like distribution of dye aggregates within the polymer sample (Figure 1 and SI Figure S13). This is attributed to the long-range alignment of BBON’s planar aromatic core, thus enabling long-range stacking of the molecules and potential quenching of emission.^29^ BMBE samples were found to photobleach at the lowest laser power with little evidence of aggregate substructures (SI Figure S14). Such sensitivity, due to BMBE’s less-stable double-bond core,^30^ could explain the limited evidence of AIEE in steady-state lifetime measurements due to photobleaching during sample processing, handling, and measurement.

The long-lived lifetime parameter (τ_2_) extracted from fluorescence lifetime measurements was found to increase with dye content in BBS-, BMBS-, and BBON-doped HDPE samples. Lifetime parameter increases were found to be highly linear in the case of BMBS. Meanwhile, τ_2_ was found to saturate above 0.05–0.07 wt % dye content in BBON-doped samples which is typical in ACQ or other long-range supramolecular ordered structures (Figure 1 and SI Figure S13). Fluorescence lifetime contributions for BMBE were undetectable below 0.07 wt % dye content for both emission wavelengths, which we attribute to a combination of reduced emission due to photobleaching and corresponding instrument sensitivity.^24^

This dye scope revealed that successful determination of AIEE requires nonplanar dyes featuring conjugated electron contributions from both double bonds and aromatic groups—such as BBS and BMBS. These two OBs, both featuring aromatic stiff nonplanar cores, gave the strongest AIEE response in HDPE. However, the weaker AIEE in BBON (due to long-range π–π-type interactions) could be exploited in applications where high loading levels have little impact on use. High dye loading levels are typically avoided—especially in food applications, due to both increased costs and regulatory oversight from the Food and Drug Administration (FDA) or analogous government bodies.

These initial hypotheses hold true for HDPE-based matrices, but in polymers with increased backbone complexity, such as poly(ethylene terephthalate) (PET), poly(methyl methacrylate) (PMMA), or similar, increased dye–polymer interactions would cause elevated aggregation thresholds and modify these rudimentary correlations.

### 2.2 Structural Impacts of Polymer on Aggregation-Induced Enhanced Emission

Modification of the host in guest–host systems impacts the aggregation in two related ways: first, by changing the number of dye–polymer interactions, and second, by modifying dye solubility by changing the polarity of the host system. Aggregation becomes less energetically probable with increasing dye solubility or interactions between well-dispersed dye molecules in systems.^8^

BBS contains several chemical moieties (e.g., C–H, C–O, C–N, and C_6_H_6_) that may be susceptible to hydrogen bonding, van der Waals interactions, or π–π interactions with the host polymer matrix. We originally hypothesized that increasing the polarity of the host system, such as in PET, PLA, poly(methyl methacrylate) (PMMA) or poly(ethylene terephthalate-*co*-cyclohexane dimethanol) (PET-G), would increase the aggregation threshold and reduce AIEE. Additionally, by choosing highly aromatic polymer hosts (PET, PET-G) new interactions (C–H–π and π–π) between aromatic sections in the polymer and dye could be induced, increasing dye molecular separation and further reducing thresholds.^31,32^

**Figure 3:**
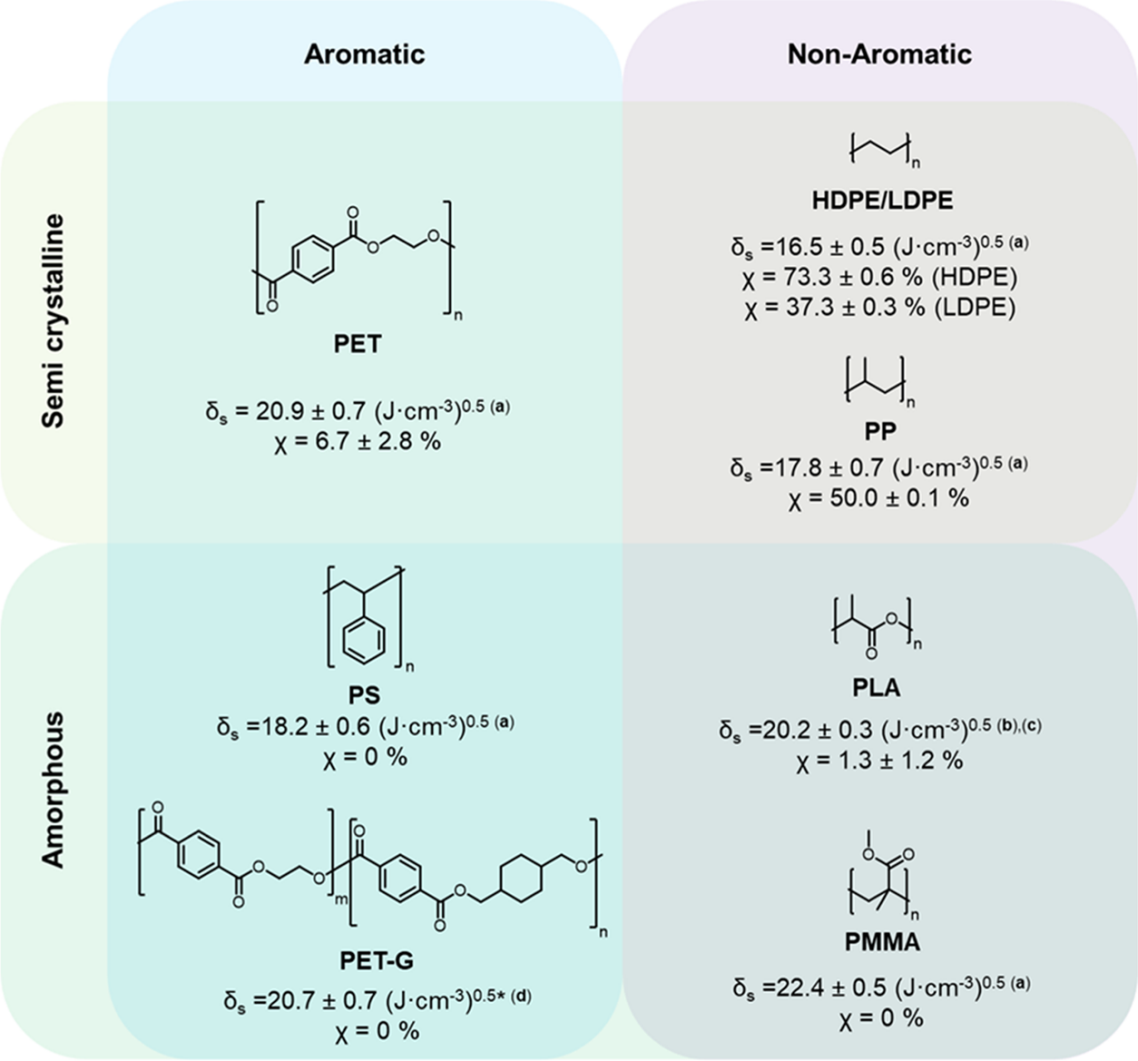
Crystallinity and solubility parameters of host polymer matrices. Host polymer systems tested for AIEE were categorized by their aromaticity, experimentally determined crystallinity (χ) through differential scanning calorimetry (DSC) (eq 4—Materials and Methods section) and reported solubility parameter (δ). Solubility parameter of PET-G calculated by average contributions from ethylene glycol terepthalate (ET) and 1,4-cyclohexane dimethanol terephthalate (CT) units (ET/CT = 2.2) from ref (35) with solubility parameter values a,^33^ b,^8^ c,^36^ and d^37^ from literature data.

Comparison between a dye and a polymer’s solubility parameter can provide rough predictions of dye solubility within a host system; solubility is maximized between two species with similar parameters.^8,33^ This method was applied to BBS and a selection of polymer hosts to estimate solubility and the resulting aggregation threshold. For small molecules, the solubility parameter can be estimated, through summation of a group’s cohesive energy contribution, *E*_coh_, and molar volume contribution, *V*_m_ (eq 2).^34^
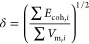
2From this calculation, using Fedors approximations, BBS′ solubility parameter is 25.82 (J/cm^3^)^0.5^ (SI Section S3 and SI Table S2).^34^ Through comparison of BBS′ and polymer’s parameters, it was predicted that BBS would show highest solubility in polar hosts such as PET-G, PET, PMMA, and PLA and lower solubility in less polar PE and PP (δ summarized in Figure 3). We hypothesized that the anticipated lower solubility would decrease the required dye concentration needed for aggregation and increase spectral intensity for any quantitative determinations.

**Figure 4:**
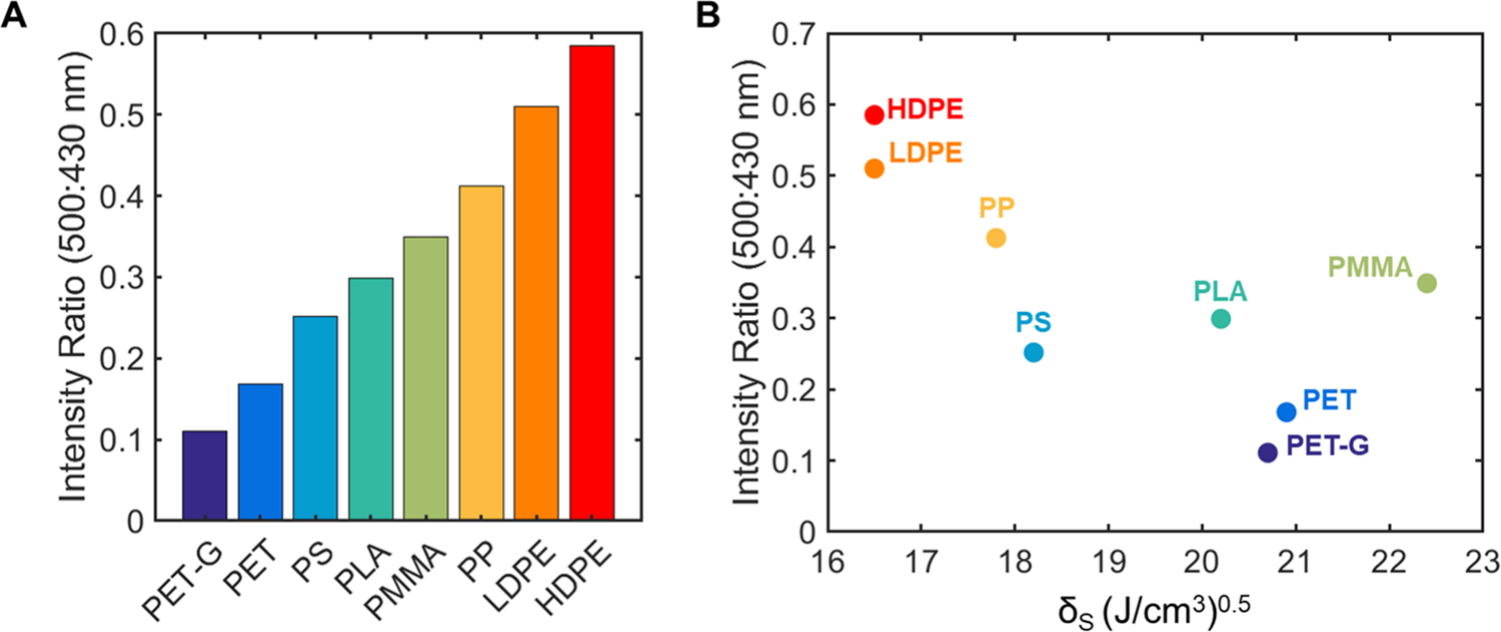
Fluorescence emission of BBS in different polymer matrices. (A) Fluorescence intensity ratio (500:430 nm) for different polymer hosts at 0.5 wt % dye loading. (B) Fluorescence intensity ratio (500:430 nm) for different polymer hosts at 0.5 wt % dye loading and their respective solubility parameters. Fluorescence intensity ratios extracted from steady-state spectra (SI Figure S16).

Loading studies (0.025–1.675 wt %) of BBS were performed in PET, PET-G, polystyrene (PS), HDPE, low-density polyethylene (LDPE), polypropylene (PP), PMMA, and PLA to compare the minimum aggregation thresholds across differing packaging plastics. As predicted through solubility parameter comparison, it was found that the aggregation thresholds were highest in the most polar systems (PET, PMMA, and PET-G) and decreased with a decrease in polymer polarity (Figure 4). Through comparison of the fluorescence intensity ratio calculated at 500/430 nm for 0.5 wt % dye loading, PET, PET-G, and PS samples displayed the lowest levels of aggregation-induced emission, attributed to both increased solubility and number of π–π or C–H–π-type interactions between the BBS and polymer main-chain aromatic groups (Figures 3 and 4).^31,32^ For example, aggregation levels in HDPE were 6-fold higher than those in PET samples at identical concentrations, which we attribute to the reduced number of interactions between BBS and structurally simple HDPE.

From these initial studies, the solubility parameter prediction fails for polymer matrices featuring aromatic groups. For example, BBS solubility in PMMA is predicted to be higher than in PS (δ = 22.4 for PMMA, δ = 18.2 for PS, and δ = 25.82 for BBS) which is suggestive of a higher aggregation threshold, but aggregation is stronger in PMMA than in PS at all concentrations tested (Figure 4). We attribute this to increases in both the number of nonaromatic and aromatic interactable moieties along the polymer backbone. With an increased number of π–π or C–H–π interactions, this aggregation threshold increases and AIEE is limited.^21^ This is further corroborated by all aromatic polymers displaying the lowest evidence of AIEE at the 500/430 nm ratio, irrespective of their calculated solubility parameters (Figure 4).

The data are also suggestive that AIEE can be impacted by steric contributions; this is most apparent for PMMA showing higher AIEE than PLA despite having a higher solubility parameter. This discrepancy could be explained by PMMA’s increased 3D structural complexity (tacticity-dependent)^38,39^—relative to PLA—providing an additional steric barrier to dye–polymer interactions. Following this steric hindrance, the system could then favor dye–dye interactions.

To understand how these differences in AIEE might translate to industrially relevant concentrations, the starting BBS-polymer concentrations were set to 0.1 and 0.5 wt % and subsequently diluted by up to 10-fold (0.01–0.1 or 0.05–0.5 wt %, respectively). No significant evidence of AIEE was detected for scaled-up tests completed with starting MB concentrations of 0.5 wt % BBS loading in aromatic polymers PET and PET-G (Table 2 and SI Table S4). Unlike the other aromatics tested, BBS-PS tests (0.5 wt %) displayed AIEE character, suggestive of a lower number of interactions between chain and dye molecule (Table 2). PS′ pendant aromatic groups, unlike those embedded along the polymer backbone in PET and PET-G (Figure 3), can have varying orientations depending on the tacticity of the polymer.^37^ This tacticity, which is also related to the flexibility of the polymer chain, could potentially disrupt π–π or C–H–π interactions between the dye and polymer, allowing higher aggregation of the BBS molecules relative to the other aromatic systems.

**Table 2:** Scaled Testing Performed with BBS and a Variety of Host Polymer Matrices^a^

polymer sample	BBS concentration (wt %)	gradient—∇ (470:430 nm)	gradient—∇ (500:430 nm)
PP	0.1	4.20	34.0
HDPE	0.1	2.97	3.30
LDPE	0.1	2.58	2.06
HDPE	0.025	0.56	0.50
PMMA	0.1	0.44	0.19
PLA	0.1	0.29	0.14
PS	0.1	0.28	0.09
PET	0.1		
PET-G	0.1		

a Tabulated gradients from 1st-order polynomial fits of fluorescence emission intensity ratios for diluted MBs. Ratios were taken between dimeric and monomeric emissions across multiple polymer hosts. Fits were produced using the MATLAB curve fitting toolbox.

Clear evidence of AIEE was detected in BBS-doped PE, PP, PLA, and PMMA samples, evidenced by enhanced emission at 470 and 500 nm with increased concentration (Table 2 and SI Table S4). The “strength” of AIEE of these systems (gradient comparison {∇}—Section S3.1), reveals that the highest levels of aggregation are detected in the systems with least polar character such as PE or PP. This is likely due to the strength of dye–dye interactions relative to the van der Waals of dye–polymer interactions. With increasing solubility parameter and backbone complexity, such as PMMA and PLA compared to the polyolefins, the strength of AIEE is found to decrease significantly (Table 2). The aggregachromic response of dilutions of a 0.1 wt % PLA is comparable to those performed in HDPE at a lower BBS loading of 0.025 wt %, highlighting the importance of host structure in AIEE. As opposed to the loading studies described above, PP displayed higher AIEE than PE (Table 2). This discrepancy is roughly attributed to differences in mixing efficacy, either due to scale-up or due to a difference in melt flow index (MFI) (25 g/min for PP versus 0.5 g/min for HDPE {Section S5—Experimental Section}).

Changes in aggregate appearance across different polymer hosts were visualized through use of confocal microscopy. In HDPE, LDPE, PLA, PMMA, and PP samples, aggregates were detectable and separable from the background fluorescence corresponding to the monomeric version of BBS (SI Figures S18–S21).^1^ The presence of aggregates is most visible among the polyolefins and less distinct in PMMA and PLA. In more polar and aromatic PET, PET-G, and PS, background fluorescence from monomeric BBS is easily detectable (detection range 400–480 nm) but no aggregates are detected when the detection range is increased to the wavelengths corresponding to excimer formation (>500 nm) due to higher aggregation thresholds.

This study confirms that the relative solubility of the BBS within the polymer matrix can be used as a crude estimate of the strength of the AIEE. However, the number of aromatic groups in the host matrix, most obviously when examining AIEE in PET and PET-G, has a greater overarching impact on the final aggregation and fluorescence behavior within the sample. As the calculation of solubility remains only a rough predictor of AIEE, a thorough understanding of the impacts of guest–host interactions can be used to maximize fluorescence response for use in quantitative analyses.

### 2.3 Crystallinity Impact on Aggregation-Induced Enhanced Emission

Dye molecules habitually reside within amorphous regions within their host polymers.^40^ The local concentration of dye can be envisioned as being dependent on the crystalline domain size. In systems with high crystallinity, the dye migrates away from the crystalline domain into the small amorphous regions, increasing the local dye concentration and promoting dye aggregation. In systems with a low crystallinity and high amorphicity, dye molecules are well dispersed within amorphous regions and are less likely to form aggregated structures. This diminished aggregachromic behavior was evident across loading studies and scaled-up testing in samples with low crystallinities such as PET, PET-G, PMMA, or PLA when compared to systems with higher crystallinities such as PP or HDPE (Figures 3 and 4, Table 2, and SI Table S4).

Additionally, this effect manifests in recycled simulations of HDPE and LDPE at identical concentrations (Figure 4 and Table 2). HDPE features little to no branching compared to LDPE, which facilitates crystalline packing and results in higher crystallinity values [37.3% {LDPE} versus 73.3% {HDPE} Figure 3]. The intensity ratio for 0.5 wt % samples of PE at 500/430 nm drops from 0.59 to 0.51 between HDPE and LDPE, which corresponds to a 40% drop in crystallinity. This is also observed in tests of HDPE and LDPE; the gradient of the 500/430 nm fit decreases from ∇ = 3.30 for HDPE to 2.06 for LDPE (Table 2). This reduction in AIEE intensity is ascribed to the drop in crystallinity as LDPE and HDPE backbones are chemically equivalent. However, the two grades of PE tested stem from different manufacturers, featuring different molecular weights and additive packages, which may affect the threshold concentrations required for AIEE.

In systems with tunable crystallinities, the relative dye concentration and aggregation probability can be altered.^12,41^ PLA and PET are polymer systems that display crystallinity flexibility. Their crystallinities can be modulated from low values (∼5%) up to moderate crystallinities (ca. 30–40%) upon annealing at temperatures above their polymer glass transition (*T*_g_ ∼ 75 °C {PET} and ∼50 °C {PLA}), promoting nucleation and growth of crystalline domains. Through our previous studies of PET, fluorescence intensity ratios stemming from measurements of diluted MB samples were only detected in annealed samples, corresponding to crystallinity increases from ∼7 to ∼30%.^1^ To monitor and compare the evolution of AIEE behavior with increasing crystallinity across aromatic and nonaromatic host systems, PET and PLA samples were subject to cumulative annealing processes at 100 and 80 °C, respectively.

PET-G remains fully amorphous during annealing unless under specific conditions (SI Figure S22), and was used to verify that changes in fluorescence were primarily crystallinity-induced.^42^ No change in fluorescence ratios or crystallinity was seen between pre- and post-anneal (60 °C) PET-G samples (SI Figure S22). This annealing study confirmed that annealing of samples caused minimal dye migration and fluorescence enhancements unrelated to crystallinity changes.

Starting masterbatch concentrations of 0.1 wt % (PLA) and 0.5 wt % (PET) BBS loading (relative to polymer matrix) were chosen to ensure that changes in AIEE were observable after annealing above the polymer glass transition temperatures. To investigate the effect of the annealing process on the AIEE behavior of the PLA system, the fluorescence intensity and appearance of PLA samples were recorded during progressive anneals (100 °C). Through a cumulative 90 min anneal time, the crystallinity increased from 3.5 to 27.2%, corresponding to the growth of crystalline domains (calculated by DSC according to eq 4 {Materials and Methods section} and SI Table S5). The gradient of the calculated fluorescence intensity ratios fit (eq 1) was observed to increase by 1.3-fold at 470/430 nm and by 2.4-fold at 500/430 nm (SI Figure S23). This increase in the strength of AIEE behavior was attributed to the increase in the number of aggregates within the system with increasing anneal time—confirmed using confocal microscopy (SI Figure S24). Digital photographs of samples fluorescing under UV light revealed a red shift in emitted color with increased crystallinity (SI Figure S23). Additionally, this change in color was accompanied by an increase in sample opacity, as is expected from increased crystallinity. We postulate that these changes are entirely due to the increase in crystallinity forcing BBS into the amorphous region and increasing its local concentration, driving aggregation.

**Figure 5:**
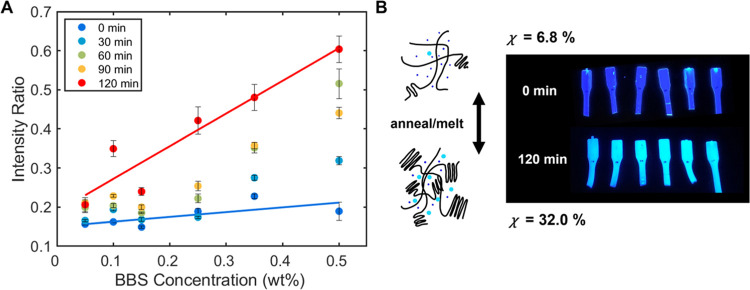
Crystallinity effect of AIEE in BBS-PET samples. (A) Fluorescence intensity ratio (500:430 nm) with increasing concentration at different time points during a 100 °C annealing process. Fitting was performed using the MATLAB curve fitting toolbox. Error bars represent the standard error from annealing and testing 5 samples per concentration from the same batch. (B) Schematic representation of morphological changes during the annealing process, digital photographs of BBS-PET samples illuminated by 365 nm, and corresponding crystallinity values (χ) pre- and post-annealing process.

In equivalent studies of dilutions of PET 0.5 wt % MBs, similar increases in the strength of AIEE were observed with increasing annealing time (Figure 5). The gradients of intensity ratios were found to increase at both emission wavelengths (1.9-fold at 470/430 nm and 3.2-fold at 500/430 nm) when the sample crystallinity was increased from 6.8 to 32.0% (Figure 5 and SI Table S6). Similar to PLA, digital photographs of PET reveal a color and opacity change with increasing crystallinity, as crystalline growth promotes aggregation of the BBS molecules and red-shifting of emission. Nonlinearity of the intensity ratios was observed at intermediate anneal times, which is ascribed to heating inhomogeneity during the annealing process. Confocal microscope images taken of PET-BBS samples preanneal revealed no evidence of BBS aggregation but upon annealing, small aggregates began to appear which increased in density during the annealing process (SI Figure S25).

Notably, the increases in aggregation ratios (3.2-fold {PET} and 2.4-fold {PLA} at 500/430 nm) and crystallinity at the maximum time point (27.2 ± 0.6% {PLA} and 32.0 ± 0.3% {PET}) were similar in value for both polymers. Yet, the starting concentration of PET (0.5 wt %) was 5 times higher than that of the PLA (0.1 wt %) masterbatch. This 20-fold decrease in AIEE sensitivity observed for PET is largely attributed to differences in the aggregation threshold for aromatic systems (Figure 4 and Table 2). PLA and PET both feature ester bonds which contain oxygen atoms, which can interact with the BBS molecule to increase the number of interactions. However, polymer–dye π-type interactions due to aromaticity were previously highlighted to govern the final AIEE of the system.

Crystallinity is a major factor in the final AIEE of the system. Systems with increased crystalline character display higher AIEE levels due to high local dye concentrations within the amorphous domains. In annealed systems, such as PET and PLA, the aggregation threshold can be decreased, potentially enabling lower dye loadings within highly aromatic polymers.

## 3 Conclusions

Here, through use of fluorescence-based characterization and confocal microscopy, we show that AIEE largely depends on the threshold aggregation concentration. This threshold is largely governed by the number of interactions between chosen dye (guest) and polymer (host), where minimizing these intermolecular interactions facilitates aggregation. Additionally, thresholds are impacted by a host polymer matrices’ crystallinity, aromatic groups, and solubility parameters. We successfully determine that the rigid and aromatic BBS fluorophore core and its methyl-substituted analogue, BMBS, show the highest potential for the quantitative determination of AIEE in plastics and related applications. This increased sensitivity to aggregachromism relative to the other dyes screened was attributed to minimal guest–host interactions, favoring dye–dye interactions, and increasing the ability to detect AIEE.

Moreover, we demonstrate that similar changes in dye aggregation can be realized through change of host matrix. The solubility parameter of various polymer hosts allowed a crude prediction of AIEE with highest efficacy in low-polarity hosts, such as PE and PP, and lowest in highly polar hosts, such as PLA, PMMA, and PET. These findings were consolidated using fluorescence testing, while the effects of steric bulk and aromaticity were demonstrated to impart secondary effects on AIEE. The presence of aromatic groups—most notably in PET and PET-G systems, was linked to the highest aggregation thresholds, owing to a predicted elevation of guest–host interactions (π–π or C–H–π) between dye and polymer. We established that high aggregation threshold levels can be surmounted by increasing the crystallinity of the system. Annealing of PET up to 32% crystallinity (from ∼7%) increases the strength of AIEE by 3.2-fold (500/470 nm) and was accompanied by the growth of aggregates detected via confocal microscopy. This is also replicated in nonaromatic annealable PLA where AIEE was increased by 2.4-fold (500/470 nm) through annealing and was similarly paired with the appearance of aggregates with increasing crystallinity (∼5 to ∼30%). Similar increases in aggregation strength with comparable crystallinities of PET and PLA required vastly different starting masterbatch concentrations (0.1 wt % for PLA vs 0.5 wt % for PET) due to the enhanced solubility of BBS in PET matrices. Following these findings, we propose that the aggregation of BBS within a given host is primarily governed by the presence of aromaticity, followed by crystallinity and then polarity of the host system.

Understanding and predicting how different polymer systems respond to fluorescence dyeing becomes vital with the emerging number of new biobased, biodegradable, or alternative plastic materials on the market. Additionally, this work suggests that AIEE can be tailored to specific sectors through dye choice; for example, choosing a low-sensitivity dye (e.g., BBON) for applications that can withstand higher dye loadings. Examining the intricate dynamics of aggregachromic guest–host interactions, our findings facilitate the development of advanced polymer systems tailored for applications ranging from recycled content assessment to precise process monitoring and beyond.

## 4 Materials and Methods

### 4.1 Materials

Polymer pellets were purchased from Hardie Polymers. Food-grade Sabic HDPE B624LS has an MFI of 0.5 dg/min at 190 °C/2.16 kg and a quoted melting point of 135 °C. PET was Ramapet N1 polymer pellets, manufactured by Indorama Ventures, with a quoted melting point of 247 ± 2 °C and of extrusion grade ([η] = 0.80 ± 2 dL/g). Carmel Olefins Ltd. manufactured PP Capilene T 89 E has an MFI of 25 g/10 min at 230 °C/2.16 kg and a quoted vicat softening temperature of 153 °C. PLA, Luminy LX575 manufactured by Total Corbion, has a quoted melting temperature of 165 °C and a glass transition temperature of 60 °C. PET-G was supplied by Push Plastic, with a quoted vicat softening temperature of 85 °C. Polystyrene crystal 1540 manufactured by Total Petrochemicals & Refining USA, Inc., has a Melt flow index (200 °C/5 kg) of 12 g/10 min and vicat softening point (10N) of 91 °C. LDPE Lupolen 2420 H manufactured by LyondellBasell Industries has a Melt flow index (190 °C/2.16 kg) of 1.9 g/10 min and vicat softening point of 94 °C. PMMA PLEXIGLAS 8N was manufactured by Evonik Industries AG has a Melt flow index (230 °C/3.8 kg) of 3 g/10 min and vicat softening point of 108 °C.

All optical brighteners were used as received unless specified otherwise. 1,4-Bis(benzo[*d*]oxazol-2-yl)naphthalene (BBON), trade name Hostalux KCB and 4,4-bis(2-methoxystyryl)biphenyl (BMSB), trade name Uvitex FP, were supplied by Fluorochem. 4,4′-Bis(2-benzoxazolyl) stilbene (BBS), also known as Fluorescent Brightener 393 or Rylux OB-1, 1,2-bis(5-methyl-2-benzoxazolyl)ethylene (BMBE), and 4-(2-benzoxazolyl)-4′-(5-methyl-2-benzoxazolyl)stilbene (BMBS), known as Hostalux KSN, were supplied by Tokyo Chemical Industry UK. 2,5-Bis[5-(*tert*-butyl)-1,3-benzoxazol-2-yl]thiophene (BTBBT), also known as Uvitex or Tinopal OB, was supplied by Apollo Scientific.

### 4.2 Sample Preparation

PET, PLA, and PET-G pellets were dried in a Fistreem vacuum oven fitted with an Edwards RV5 vacuum pump at 120, 60, and 60 °C, respectively, for 16 h to prevent degradation during extrusion. Polyolefins were not dried preprocessing.

HDPE-OB (BBS, BMBS, BMSB, BBON, BMBE, and BTBBT) samples were prepared by dispersing OB in HDPE at 200 °C (0.1–1 wt % wrt HDPE matrix) in a HAAKE Minilab conical twin-screw micro compounder. Samples were cooled in a water bath and dried under vacuum before further processing.

Polymer–dye master-batches were prepared by melt-blending optical brighteners (doses ∼2.5 g) with the chosen host matrices at 100 rpm and 280 °C (PET), 260 °C (PET-G), and 200 °C (PLA, HDPE, PP, PS, PMMA, LDPE) in a HAAKE Process 11 corotating twin-screw extruder or using a HAAKE Minilab conical twin-screw micro compounder. Samples were pelletized postprocessing and dried under vacuum. Scaled-up tests were performed by diluting MBs in a HAAKE Polylab 16 to dilute the original MBs to 0.01–0.1%. Samples were processed at 100 rpm, 200 °C (PLA, HDPE, PP, PS, PMMA, LDPE), 260 °C (PET-G), or 280 °C (PET) and cooled through a water bath before pelletizing. Samples were dried before further processing.

### 4.3 Dumbbell Preparation

Dye–polymer pellets (0.01–1 wt %) were injection molded in a Minijet II pro micro piston injection molder to match ISO 527-2-1BA. Cylinder temperatures were set to 200–210 °C (HDPE, PP, PLA, LDPE, PMMA, and PS), 260 °C (PET-G), or 280 °C (PET) and mold temperatures of 60 °C (HDPE, PS, PLA, LDPE, PMMA, PP) or 80 °C (PET and PET-G), injection pressures of 300–600 bar for 5 s, and a postinjection pressure of 100–300 bar for 5 s.

### 4.4 Fluorescence Emission Spectra

Fluorescence intensity measurements were conducted on a Cary Eclipse Fluorescence Spectrophotometer from Agilent paired with the Cary Eclipse Software. Emission spectra were obtained by exciting dumbbell samples at 325 nm using slit widths of 2.5 mm for outgoing and incoming beams and measuring emission from 350 to 600 nm. All resulting spectra were normalized either manually or via the Felix software to minimize sample discrepancies. Five samples of each sample batch were produced, and errors were calculated by dividing standard deviation by the square root of sample number.

### 4.5 Fluorescence Lifetimes

Fluorescence lifetime measurements were recorded on an Edinburgh Instruments F900 instrument paired with F900 software. A 340 nm picosecond pulsed LED, 500 ns pulse period, was chosen as the excitation source, and lifetimes were recorded over a 200 ns period. The resulting multiexponential decays were deconvoluted and fitted using nonlinear lest-square fitting on the F900 software (eq 3). Three repeats per sample batch were measured unless specified otherwise. Errors were calculated by dividing standard deviation by the square root of sample number.
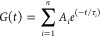
3where *A_i_* represents decay amplitude, τ_*i*_ represents the lifetime parameter, and *t* is time.

### 4.6 Confocal Microscopy

Confocal microscopy was performed on a Leica SP8 confocal microscope. Imaging was performed using a 405 nm laser, a 63 × 1.20 numerical aperture oil immersion objective, and a laser scan speed of 100 Hz, pinhole aperture set to 1.0 Airy. Images recorded were 1024 pixels × 1024 pixels and recorded across the entirety of the sample. Polymer samples were loaded onto microscope slides and covered with a glass coverslip.

### 4.7 Differential Scanning Calorimetry

DSC was performed using a TA Instruments DSC 2500. Samples weighing between 3 and 10 mg were loaded into preweighed *T*_zero_ pans and lids. Samples were subjected to heat–cool–heat with thermal ramps at 10 °C/min up to 250 (HDPE and PP) or 300 (PET) and cooled to 0 or −80 °C at cooling rates of 5 °C/min. Three repeat runs were performed for each sample unless stated otherwise. System was purged with nitrogen at 50 mL/min. Analyses were performed on the TRIOS software. Sample crystallinity was calculated according to eq 4, where Δ*H*_m_ and Δ*H*_c_ are the melting and cold-crystallization enthalpies, respectively, and Δ*H*_m_^°^ is the melting enthalpy of perfectly crystalline HDPE (293 J/g), PP (207 J/g), PLA (93 J/g), and PET (140 J/g).
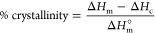
4

### 4.8 Annealing Studies

Samples were annealed in a Fistreem vacuum oven at either 120 °C (PET) or 80 °C (PLA). Samples were allowed to cool to room temperature slowly. The resulting crystallinity was measured by DSC and calculated using eq 4.

### 4.9 Solubility Parameter Calculation

Solubility parameters were calculated using the summation of cohesive energy contributions (∑*E*_coh,*i*_) and molar volumes (∑*V*_m,*i*_) from molecular motifs according to eq 5. Fedors literature values for *E*_coh_ and *V*_m_ were taken from refs (31,32) due to breadth of data availability.
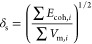
5

## Supporting Information Available

Further experimental details, measurements, and data including fluorescence lifetime measurements, confocal microscopy, and host modification and annealing studies (PDF)
