# Supplementary material for: Effect of Polymer Host on Aggregation-Induced Enhanced Emission of Fluorescent Optical Brighteners
Source: ACS Appl Polym Mater. 2024 Jan 31;6(3):2031–40. doi: 10.1021/acsapm.4c00090 (PMC10862481; doi:10.1021/acsapm.4c00090)
Supplement: Supplementary file 1 — ap4c00090_si_001.pdf [file ap4c00090_si_001.pdf]

Supporting Information

Effect of polymer host on aggregation induced enhanced emission of fluorescent optical brighteners

Authors: Zoé O. G. Schyns[†,‡], Thomas M. Bennett[†,‡], Gemma E. Davison[§], Michael P. Shaver[†,‡*]

[†] Department of Materials, School of Natural Sciences, University of Manchester, Manchester, M13 9BL, United Kingdom
[‡] Sustainable Materials Innovation Hub, Henry Royce Institute, University of Manchester, Manchester, M13 9BL, United Kingdom
[§] ReCon² Limited, Henry Royce Institute, University of Manchester, Manchester, M13 9BL, United Kingdom
*Corresponding author. Email: michael.shaver@manchester.ac.uk

Figures - 25

Tables - 4

Contents

1 Optical Brightener Scope 3
1.1 Fluorescence Lifetime Measurements 3
1.1.1 BMBS 4
1.1.2 BBON 5
1.1.3 BMBE 6
1.1.4 BBS 7
2 Optical Brighteners Dilutions (0.1 wt%) 8

2.1 BMBS 8

2.2 BBON 9

2.3 BMBE 9

2.4 Confocal Microscopy 10

2.4.1 BMBS 10

2.4.2 BBON 11

2.4.3 BMBE 12

2.4.4 BBS 12

3 Calculation of Solubility Parameter 13

4 Modification of Host 14

4.1 Loading studies 14

4.2 Example scaled-up dilution 15

4.3 Host Variation 16

4.4 Confocal Microscopy 17

4.4.1 LDPE 17

4.4.2 PLA 18

4.4.3 PMMA 18

4.4.4 PET-G 19

5 Annealing Studies 20

5.1 Annealing Studies on PET-G 20

5.2 PLA 21

5.2.1 Crystallinity Studies on PLA 21

5.2.2 Fluorescence 21

5.2.3 Confocal microscopy 22

5.3 PET 23

5.3.1 Crystallinity Studies on PET 23

5.3.2 Confocal microscopy 23

6 References 25

1 Optical Brightener Scope

Table S1 – Tabulated summary of the optical brighteners used within this study and their abbreviation and trade name.

Dye name	Abbrevation	Trade Name
4,4'-Bis(2-benzoxazolyl) stilbene	BBS	Fluorescent Brightener 393 or Rylux OB-1
4-(2-Benzoxazolyl)-4'-(5-methyl-2-benzoxazolyl)stilbene	BMBS	Hostalux KSN
1,4-Bis(benzo[d]oxazol-2-yl)naphthalene	BBON	Hostalux KCB
1,2-Bis(5-methyl-2-benzoxazolyl)ethylene	BMBE	-
4,4-Bis(2-methoxystyryl)biphenyl	BMSB	Uvitex FP
2,5-Bis[5-(tert-butyl)-1,3-benzoxazol-2-yl]thiophene	BTBBT	Uvitex or Tinopal OB

1.1 Fluorescence Lifetime Measurements

Fluorescence lifetime analyses were performed on Edinburgh instruments F900 software. Through an iterative fitting process, bi-functional fluorescence decay functions produced the most accurate fit for the optical brighteners tested:

$$F(t) = A_1 e^{\frac{-t}{\tau_1}} + A_2 e^{\frac{-t}{\tau_2}} \#(S1)$$

Where A_1 and A_2 represent the decay amplitudes and τ_1 and τ_2 represent the short lived and long lived lifetime parameters and t time. Here the short-lived lifetime parameter represents the presence of the monomeric form of a dye and the long-lived parameter the presence of molecular aggregates.

1.1.1 BMBS

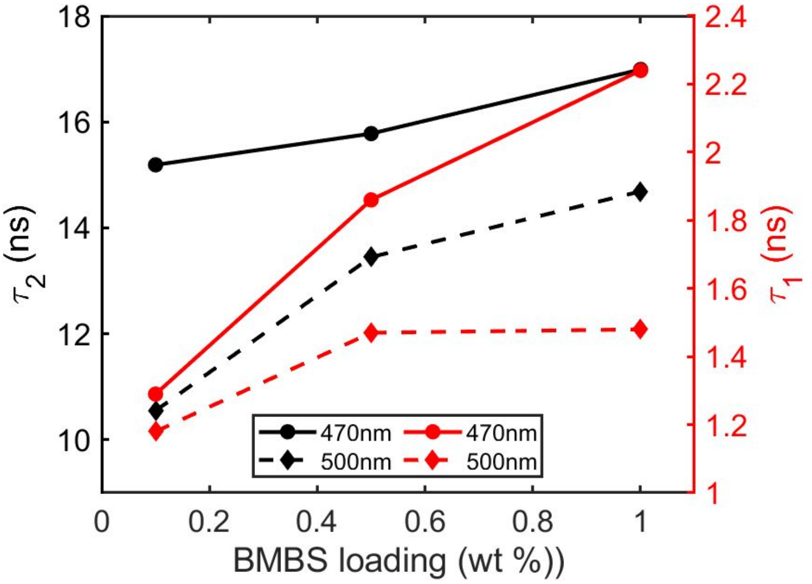

Figure S1 – Fluorescence lifetime parameters of BMBS doped HDPE samples (0.1 – 1 wt%). Lifetime parameters calculated from fluorescence lifetime decay curves according to **Equation S1**. Samples excited at 325 nm and lifetimes recorded at both 470 nm and 500 nm, τ_1 represents the short-lived component and τ_2 the long-lived component (**Section S2.2**).

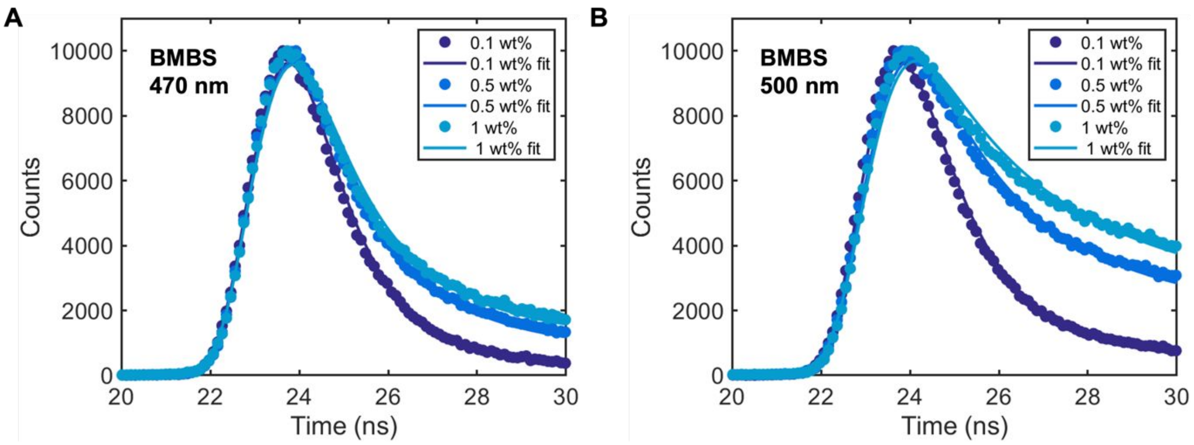

Figure S2 – Fluorescence lifetime decay curves (•) and fitted with bi-exponential decay curves (—) of BMBS doped HDPE samples (0.1 – 1 wt%). Samples excited at 325 nm and lifetimes recorded at both A) 470 nm and B) 500 nm.

1.1.2 BBON

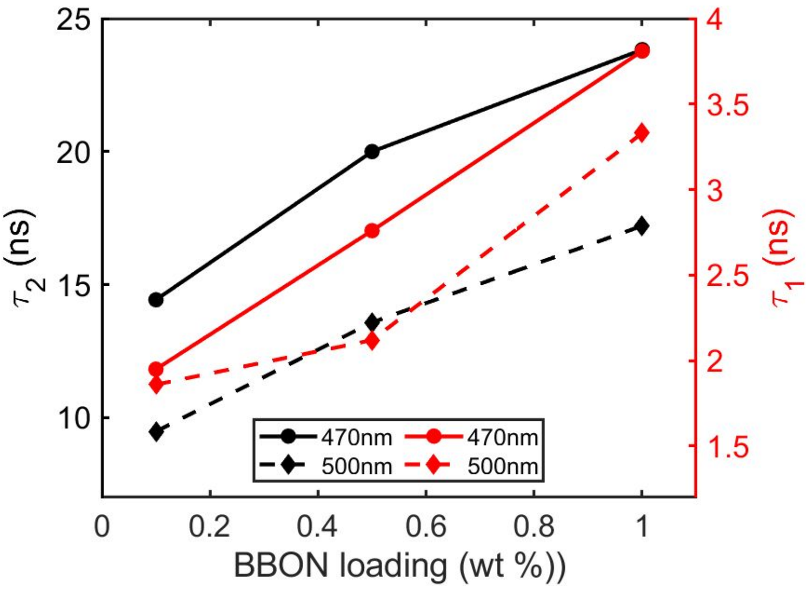

Figure S3 - Fluorescence lifetime parameters of BBON doped HDPE samples (0.1 – 1 wt%). Lifetime parameters calculated from fluorescence lifetime decay curves according to **Equation S1**. Samples excited at 325 nm and lifetimes recorded at both 470 nm and 500 nm, τ_1 represents the short-lived component and τ_2 the long-lived component (**Section S2.2**).

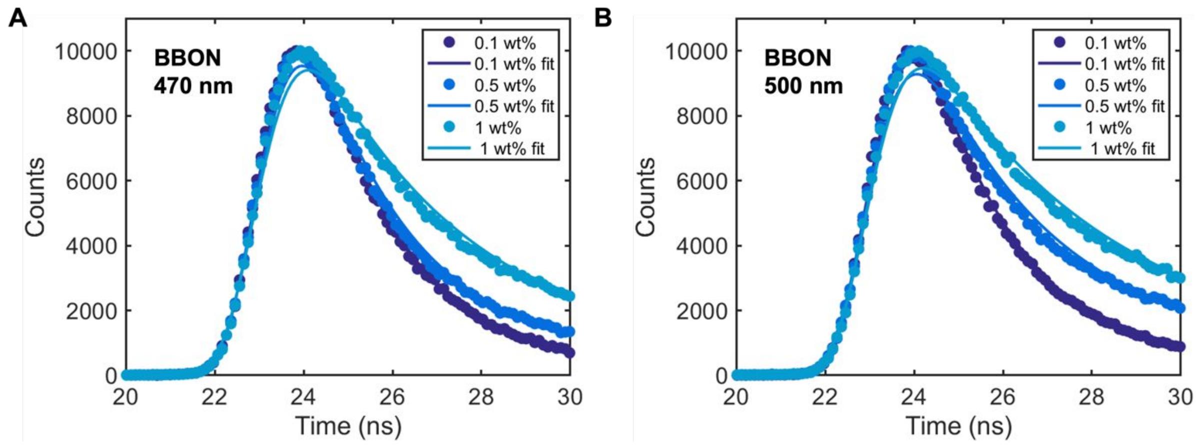

1.1.3 BMBE

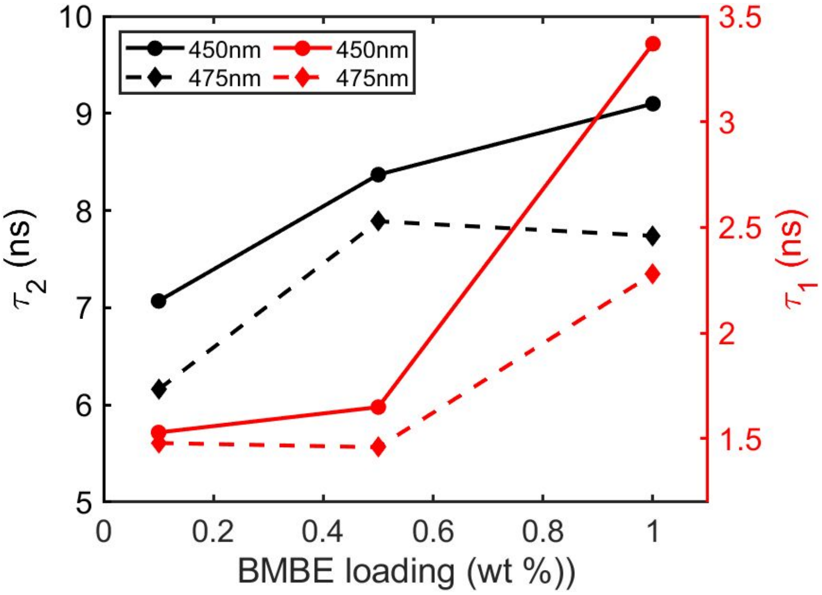

Figure S4 - Fluorescence lifetime decay curves (•) and fitted with bi-exponential decay curves (—) of BBON doped HDPE samples (0.1 – 1 wt%). Samples excited at 325 nm and lifetimes recorded at both A) 470 nm and B) 500 nm.

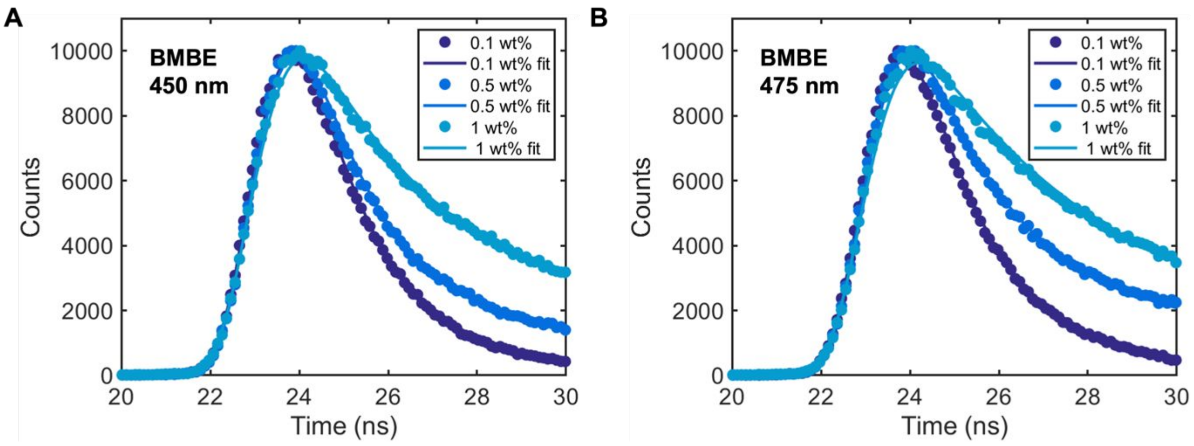

Figure S6 - Fluorescence lifetime decay curves (•) and fitted with bi-exponential decay curves (—) of BMBE doped HDPE samples (0.1 – 1 wt%). Samples excited at 325 nm and lifetimes recorded at both A) 450 nm and B) 475 nm.

1.1.4 BBS

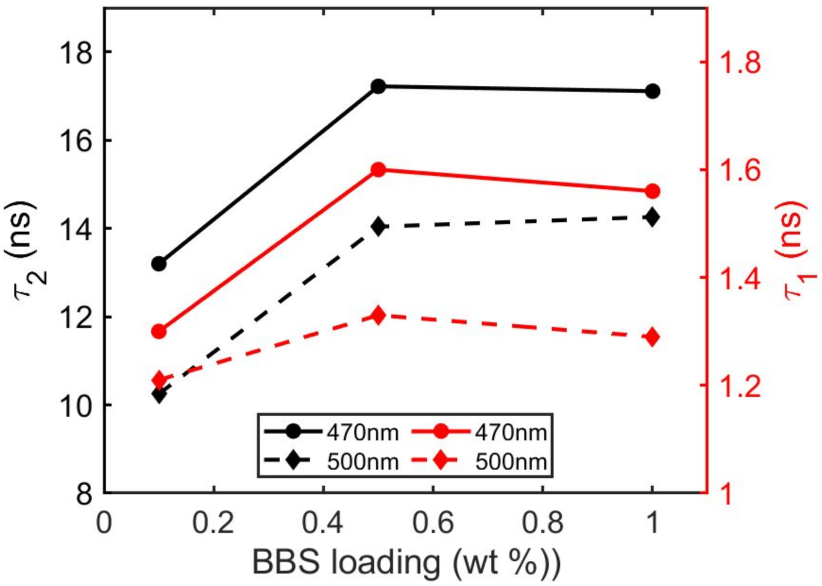

Figure S7 - Fluorescence lifetime parameters of BBS doped HDPE samples (0.1 – 1 wt%). Lifetime parameters calculated from fluorescence lifetime decay curves according to **Equation S1**. Samples excited at 325 nm and lifetimes recorded at both 470 nm and 500 nm, τ_1 represents the short-lived component and τ_2 the long-lived component (**Section S2.2**).

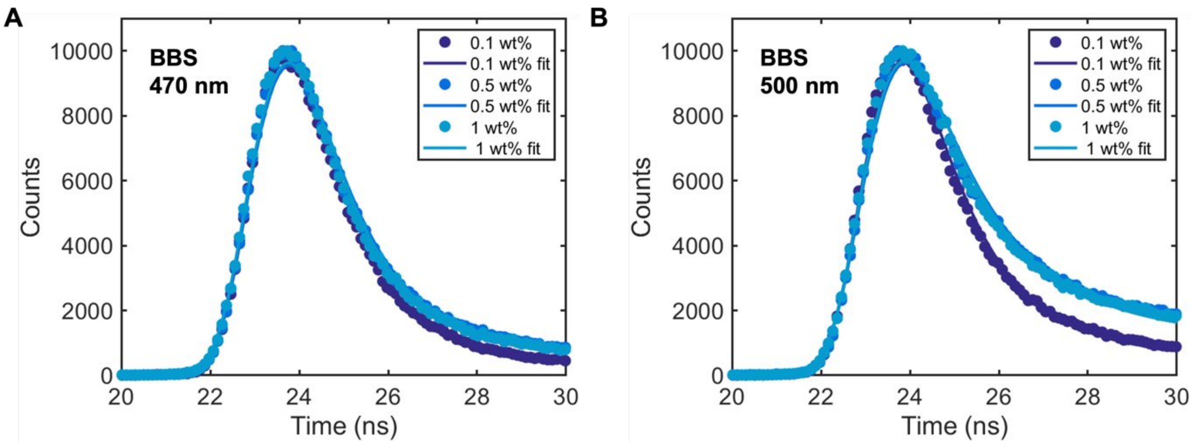

Figure S8 - Fluorescence lifetime decay curves (•) and fitted with bi-exponential decay curves (—) of BBS doped HDPE samples (0.1 – 1 wt%). Samples excited at 325 nm and lifetimes recorded at both A) 470 nm and B) 500 nm.

2 Optical Brighteners Dilutions (0.1 wt%)

Dilutions of optical brighteners at an initial masterbatch concentration of 0.1 wt% dye loading relative to polymer matrix. The fluorescence intensity ratios with increasing dye concentration were calculated using **Equation S3**:

$$Fluorescence\ Intensity\ Ratios = \frac{Intensity_{aggregate}}{Intensity_{monomer}} \#(\boldsymbol{S2})$$

Where $Intensity_{aggregate}$ corresponds to the fluorescence intensity at the peaks associated with dye aggregation (e.g.470 nm and 500 nm for dilutions of BMBS {**Figure S9**}) and $Intensity_{monomer}$ refers to the fluorescence emission maxima (~ 430 nm for BMBS {**Figure S9**})

2.1 BMBS

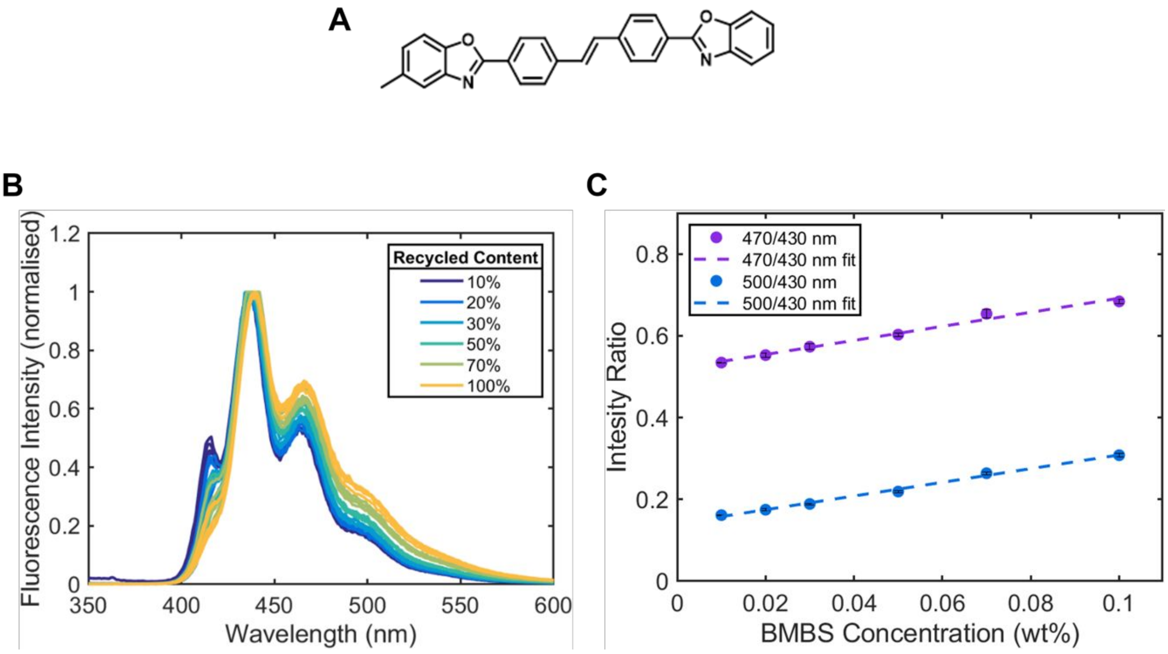

Figure S9 – **(A)** Structure of 4-(2-Benzoxazolyl)-4'-(5-methyl-2-benzoxazolyl)stilbene (BMBS) . **(B)** Fluorescence emission spectra of BMBS-HDPE samples (dilutions of 0.1 wt% relative to polymer matrix), normalised to the fluorescence emission maximum. **(C)** Intensity ratios extracted from **(B)** calculated using **Equation S2** for BMBS-HDPE.

2.2 BBON

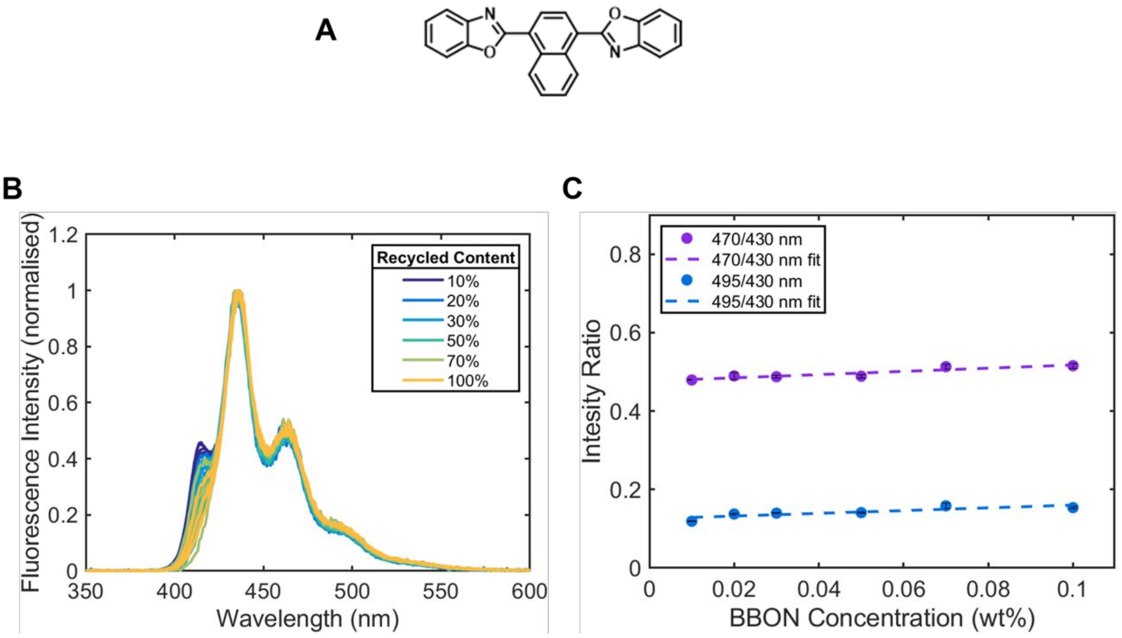

Figure S10 - **(A)** Structure of 1,4-Bis(benzo[d]oxazol-2-yl)naphthalene (BBON). **(B)** Fluorescence emission spectra of BBON-HDPE samples (dilutions of 0.1 wt% relative to polymer matrix), normalised to the fluorescence emission maximum. **(C)** Intensity ratios extracted from **(B)** calculated using **Equation S2** for BBON-HDPE.

2.3 BMBE

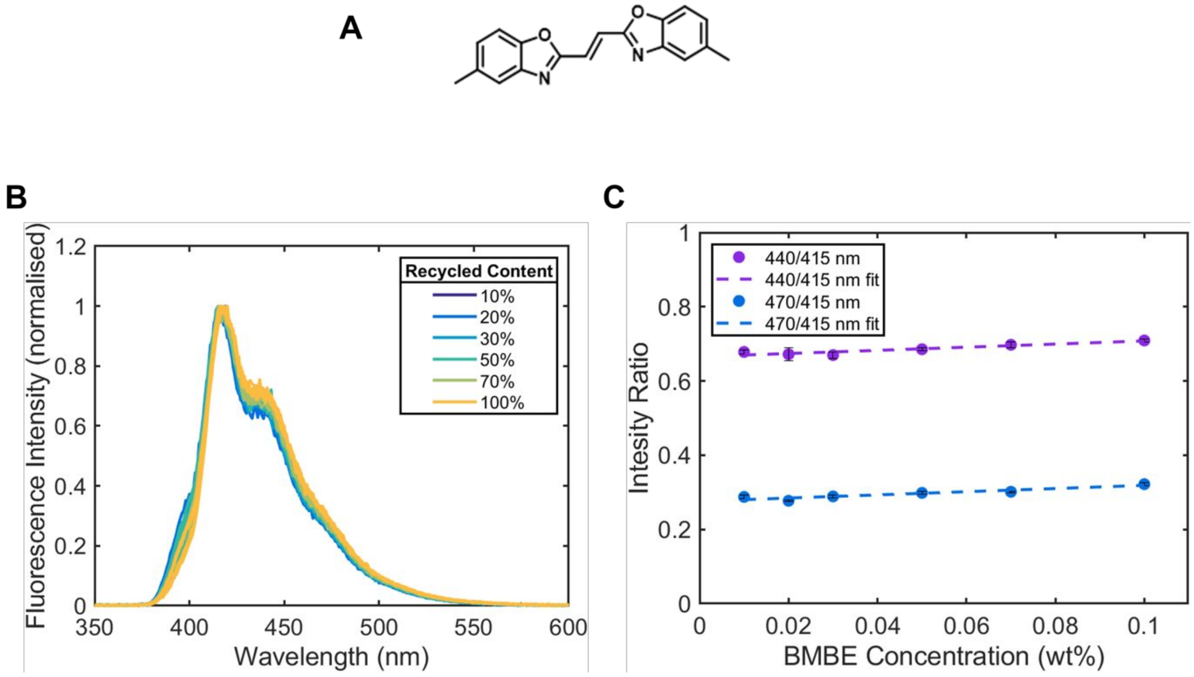

Figure S11 - **(A)** Structure of 1,2-Bis(5-methyl-2-benzoxazolyl)ethylene (BMBE). **(B)** Fluorescence emission spectra of BMBE-HDPE samples (dilutions of 0.1 wt% relative to polymer matrix), normalised to the fluorescence emission maximum. **(C)** Intensity ratios extracted from **(B)** calculated using **Equation S2** for BMBE-HDPE.

2.4 Confocal Microscopy

Confocal microscopy imaging was used to investigate aggregate substructures of optical brighteners dispersed in the HDPE matrix. The excitation wavelength was set to 405 nm for all samples and images collected between 530 – 620 nm (**Figures S12 - S15**).

2.4.1 BMBS

Aggregates present in BMBS-HDPE samples were comparable to those observed for BBS-HDPE in our previous work and below (**Figure S15**).[1]

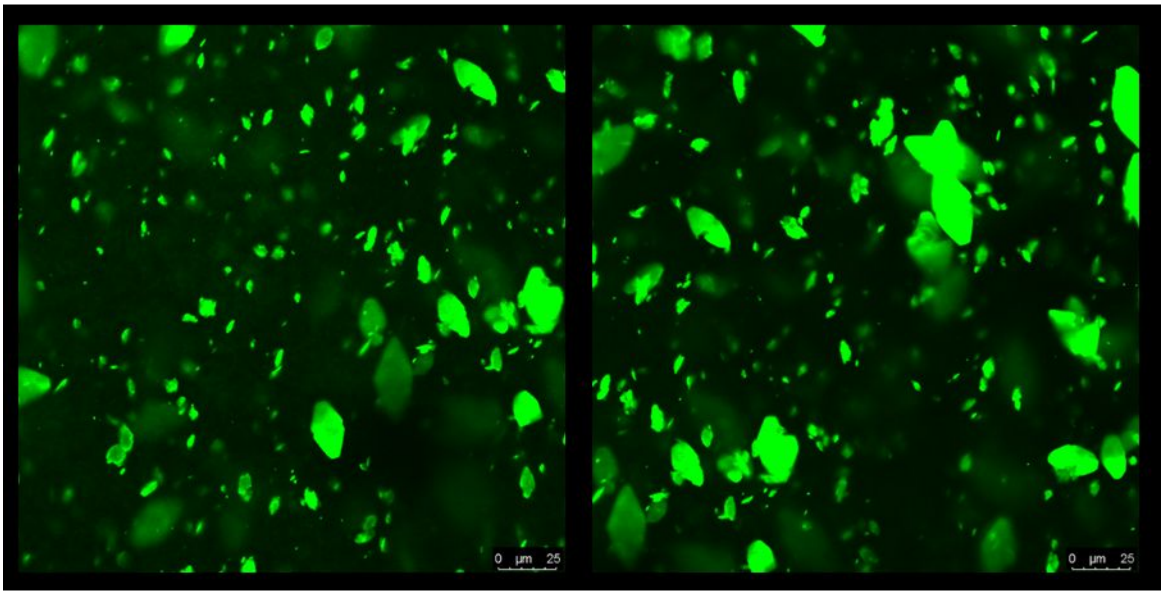

Figure S12 – Confocal images of 2.5 wt% BMBS-HDPE samples between 530-620 nm, excited by a 405 nm UV laser. Scale bars: 25 µm.

2.4.2 BBON

Aggregates formed in BBON-HDPE samples were found to be “grass-like” in appearance. This aggregate substructure can be explained by the optical brightener’s planar and aromatic structure. Structures such as these interact strongly and can pack tightly to form long-range ordered structures.

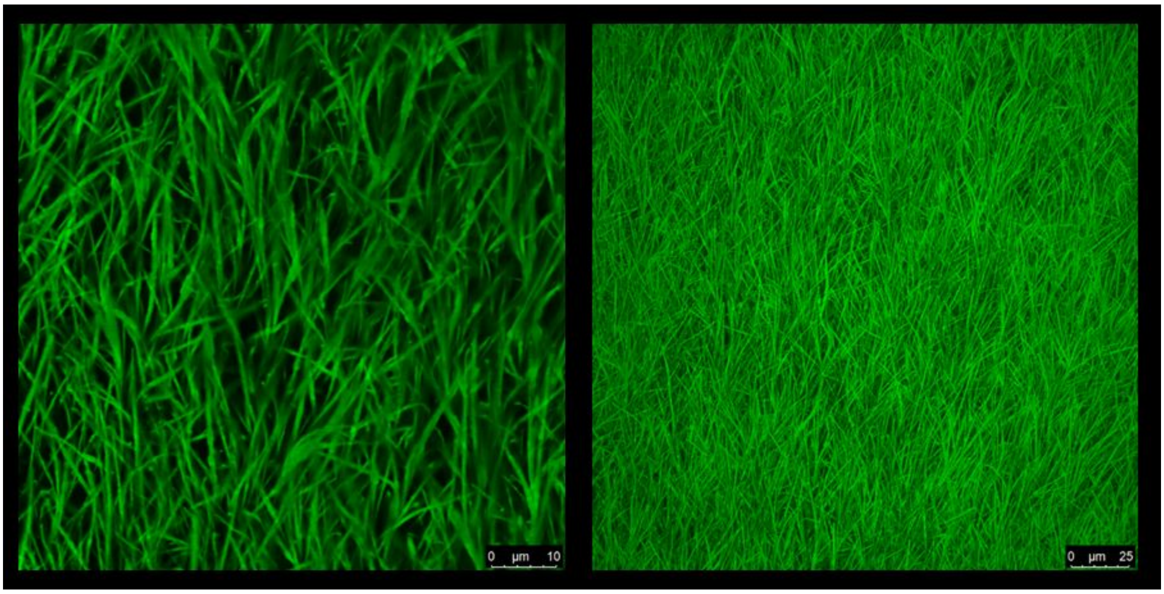

Figure S13 - Confocal images of 2.5 wt% BBON-HDPE samples between 530-620 nm, excited by a 405 nm UV laser. Left: Scale bar: 10 µm. Right: Scale bar: 25 µm.

2.4.3 BMBE

BMBE samples displayed some evidence of aggregates evidenced by the speckled background of RHS of **Figure S14**. More notably, even at the lowest laser power, the sample showed signs of photobleaching (RHS: **Figure S14**) after imaging at scan speeds of 100 Hz.

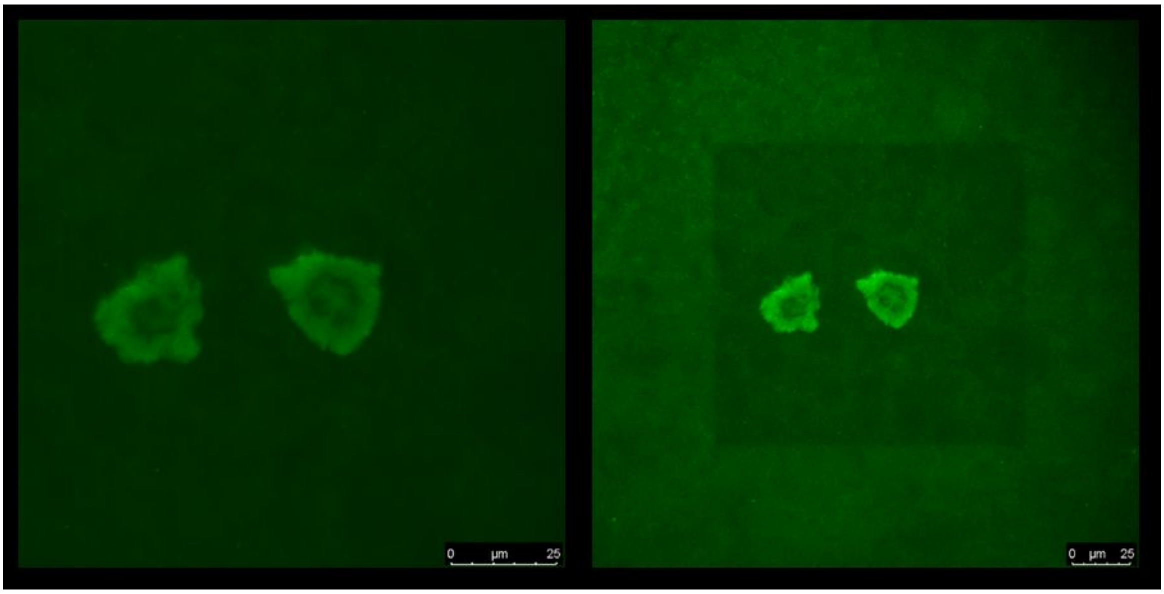

Figure S14 - Confocal images of 2.5 wt% BMBE-HDPE samples between 530-620 nm, excited by a 405 nm UV laser. Scale bars: 25 µm.

2.4.4 BBS

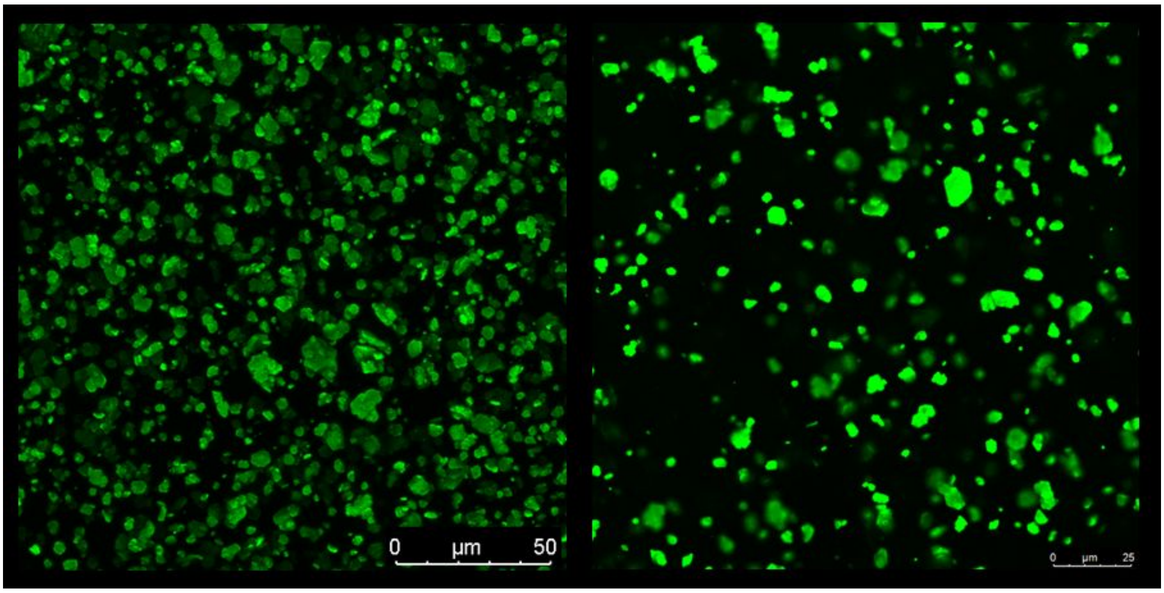

Figure S15 - Confocal images of 2.5 wt% BBS-HDPE samples between 530-620 nm, excited by a 405 nm UV laser. Left: Scale bar: 50 µm. Right: Scale bar: 25 µm. **LHS**: Figure by Zoé O. G. Schyns, Thomas M. Bennett, and Michael P. Shaver is licensed under CC BY 4.0 and was adapted with permission from all authors.[1]

3 Calculation of Solubility Parameter

The solubility parameter was calculated according to **Equation S3**:

$$\delta = \left(\frac{\sum E_{coh,i}}{\sum V_{m,\ i}}\right)^{\frac{1}{2}} \#(\boldsymbol{S3})$$

Where $\sum E_{coh,i}$ represents the summation of cohesive energy contributions and $\sum V_{m,\ i}$ represents the sum of the molar volumes from different BBS molecular features. Fedor's calculated contributions for each molecular feature were used due to breadth of data availability.[2]

Table S2 – Details of solubility parameter of BBS calculation using Equation S1. Values for E_{coh} and V_m extracted from literature.[2]

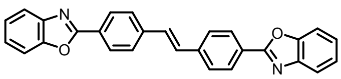<br>**Bond / Structure**	**Number**	**Σ E_{coh} (J)**	**Σ V_m (cm^3)**	**δ (J/cm^3)$^{0.5}$**
**Substituted phenyl rings**	4	4·(31940)	4·(52.4)	
**C=**	2	2·(4310)	2·(-5.5)	
**=CH-**	2	2·(4310)	2·(13.5)	
**N=**	2	2·(11715)	2·(5)	<u>25.82</u>
**-O-**	2	2·(3347)	2· (3.8)	
**5 membered closed ring**	2	2·(1046)	2·(16)	
**Conjugation in ring**	2	2·(1670)	2·(-2.2)	

Table S3 - Solubility parameters calculated for OBs using Equation S1. Values for E_{coh} and V_m extracted from literature.[2]

**Dye**	**δ (J/cm^3)$^{0.5}$**
BMBS	25.82
BTBBT	23.86
BMSB	21.83
BMBE	23.27
BBON	26.56

4 Modification of Host

Scaled tests were performed in a variety of host polymers with varying BBS loading (**Table S4**). All MBs (0.025 – 0.5 wt) were prepared using identical starting concentrated master-batches of 2.5 wt% and diluted with polymer to the appropriate starting concentrations. The "strength" of aggregation induced enhanced emission (AIEE) was estimated through comparison of the 1st order polynomial fit gradient (Example RHS: **Figure S17**) generated using MATLAB's curve fitting toolbox, calculated according to **Equation S2** and extracted from fluorescence emission spectra (Example LHS: **Figure S17**).

4.1 Loading studies

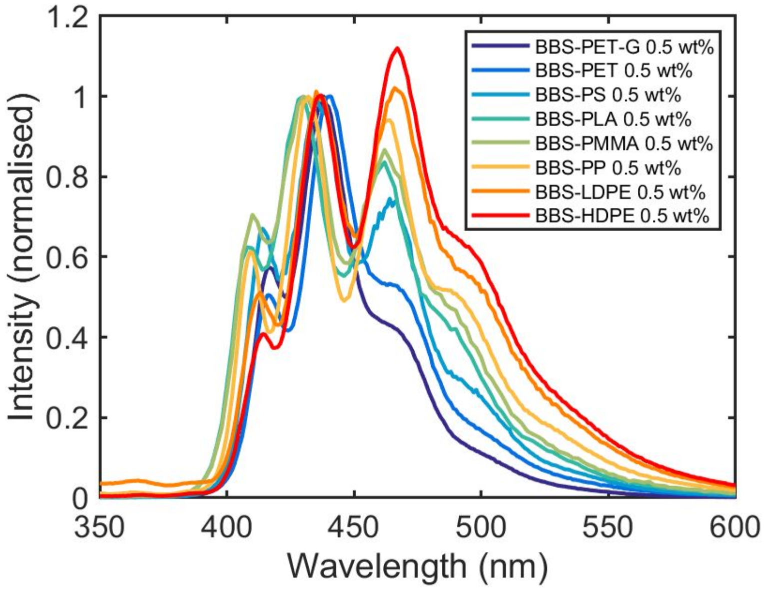

Figure S16 - (A) Fluorescence emission spectra of 0.5 wt% BBS dispersed in PET, PET-G, PS, HDPE, PLA,PP, LDPE, and PMMA measured between 350 – 600 nm with an excitation wavelength of 325 nm. All spectra normalised to the fluorescence emission maxima (present in low concentration samples) corresponding to monomer emission.

4.2 Example scaled-up dilution

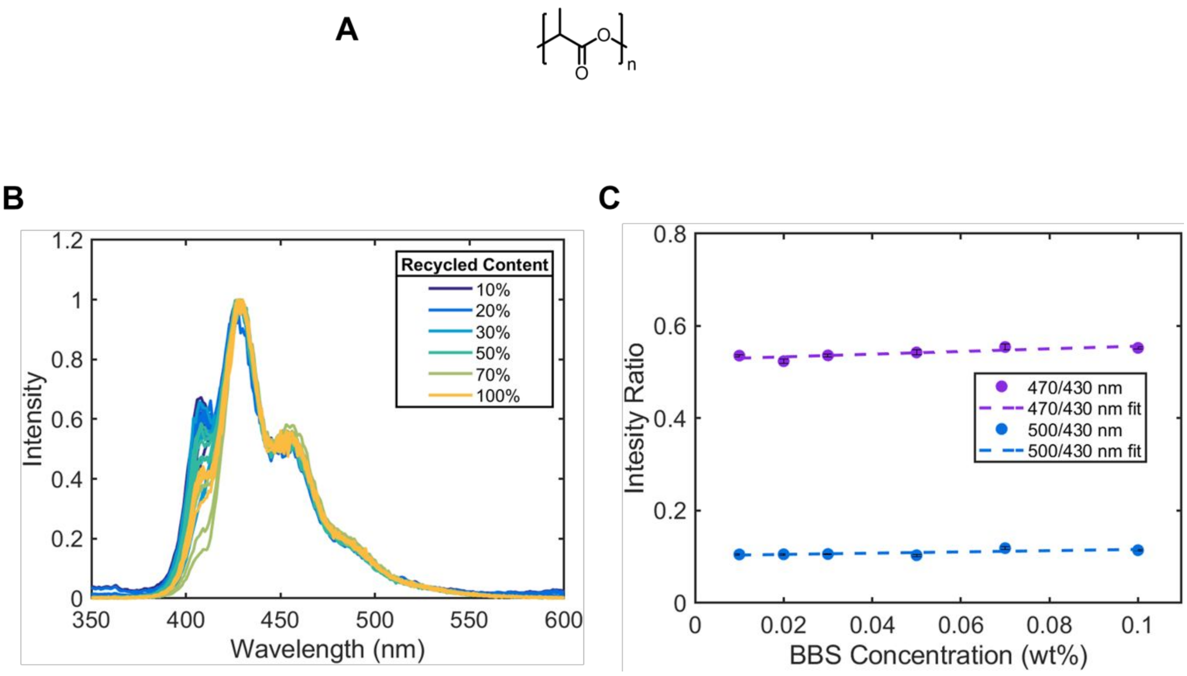

Figure S17 - (**A**) Structure of poly(lactic acid) (PLA). **(B)** Fluorescence emission spectra of BBS in PLA (dilutions of 0.1 wt% relative to polymer matrix) normalised to the monomer emission peak corresponding to the fluorescence emission maximum. **(C)** Intensity ratios calculated from **(A)** using **Equation S2** at 470 nm and 500 nm for PLA. Fits produced using the MATLAB curve fitting toolbox.

4.3 Host Variation

Table S4 - Tabulated crystallinities (χ), solubility parameters (δ) and gradients (∇) from 1st order polynomial fits of fluorescence emission intensity ratios with increasing BBS concentration in varying polymer hosts. Ratios taken between dimeric and monomeric emissions across multiple polymer hosts (example: **Figure S17**). Fits produced using the MATLAB curve fitting toolbox. Table sorted by descending 500:430nm gradient value. Samples displaying no evidence of AIEE (no concentration induced fluorescence change) had no calculable gradient. [(a)] - Values extracted from reference ([1]). Solubility parameter references: (b)[2], (c)[3], (d)[4]. (*) Solubility parameter of PET-G calculated by average contributions from ethylene glycol terepthalate (ET) and 1,4-cyclohexanedimethanol terephthalate (CT) units (ET/CT = 2.2) from reference ([4]).

**Polymer sample**	**χ (%)**	**δ (J/cm³)^0.5^**	**BBS concentration (wt%)**	**∇ Gradient (470:430 nm)**	**∇ Gradient (500:430 nm)**
**PP**	50.0 ± 0.1	17.8 ± 0.7 [(b)]	0.1	4.20 [(a)]	34.0 [(a)]
**PLA**	1.32 ± 1.2	20.2 ± 0.3 [(c)]	0.5	20.0	12.3
**HDPE**	73.3 ± 0.6	16.5 ± 0.5 [(b)]	0.1	2.97 [(a)]	3.30 [(a)]
**LDPE**	37.29 ± 0.22	16.5 ± 0.5 [(b)]	0.1	2.58	2.06
**PS**	0	18.2 ± 0.6 [(b)]	0.5	2.93	1.29
**HDPE**	73.3 ± 0.6	16.5 ± 0.5 [(b)]	0.025	0.56 [(a)]	0.50 [(a)]
**PP**	50.0 ± 0.1	17.8 ± 0.7 [(b)]	0.025	0.80	0.49
**PMMA**	0	22.4 ± 0.5 [(b)]	0.1	0.44	0.19
**PLA**	29.3	20.2 ± 0.3 [(c)]	0.1	0.29	0.14
**PS**	0	18.2 ± 0.6 [(b)]	0.1	0.28	0.09
**PET-G**	0	20.7 ± 0.8* [(c)]	0.5	-	-

**PET**	6.7 ± 2.8	20.9 ± 0.7 [(b)]	0.5	-	-
**PET**	6.7 ± 2.8	20.9 ± 0.7 [(b)]	0.1	-	-
**PET-G**	0	20.7 ± 0.8* [(c)]	0.1	-	-

4.4 Confocal Microscopy

Confocal microscopy was used to observe aggregate substructure differences across varying polymer hosts. It is important to note that little to no aggregates were detected in samples of BBS-PS, BBS-PET-G, BBS-PET within the aggregate wavelength range of 530 - 620 nm. Coincidently, these samples have little to no crystalline domains. Additionally, in other less crystalline systems such as PLA or PMMA (**Figures S19–S20**), aggregate substructures were less apparent than those more crystalline matrices (e.g. PE, PP, LDPE - **Figure S18**).

4.4.1 LDPE

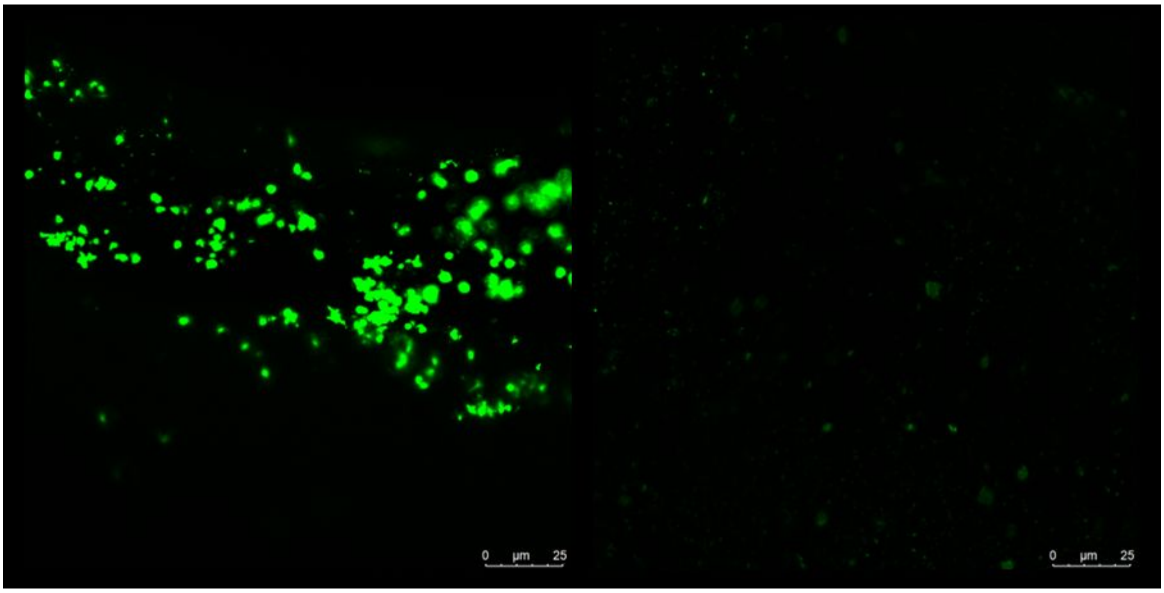

Figure S18 - Confocal images of 0.1 wt% BBS-LDPE samples between 530-620 nm, excited by a 405 nm UV laser. Scale bar: 25 µm.

4.4.2 PLA

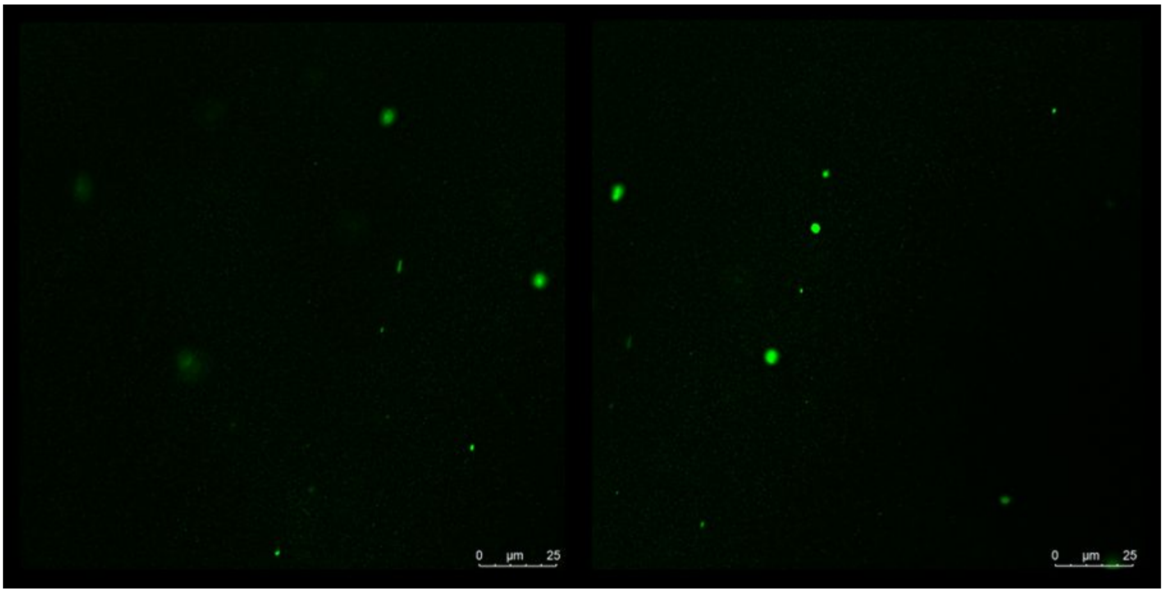

Figure S19 - Confocal images of 0.1 wt% BBS-PLA samples between 530-620 nm, excited by a 405 nm UV laser. Scale bars: 25 µm.

4.4.3 PMMA

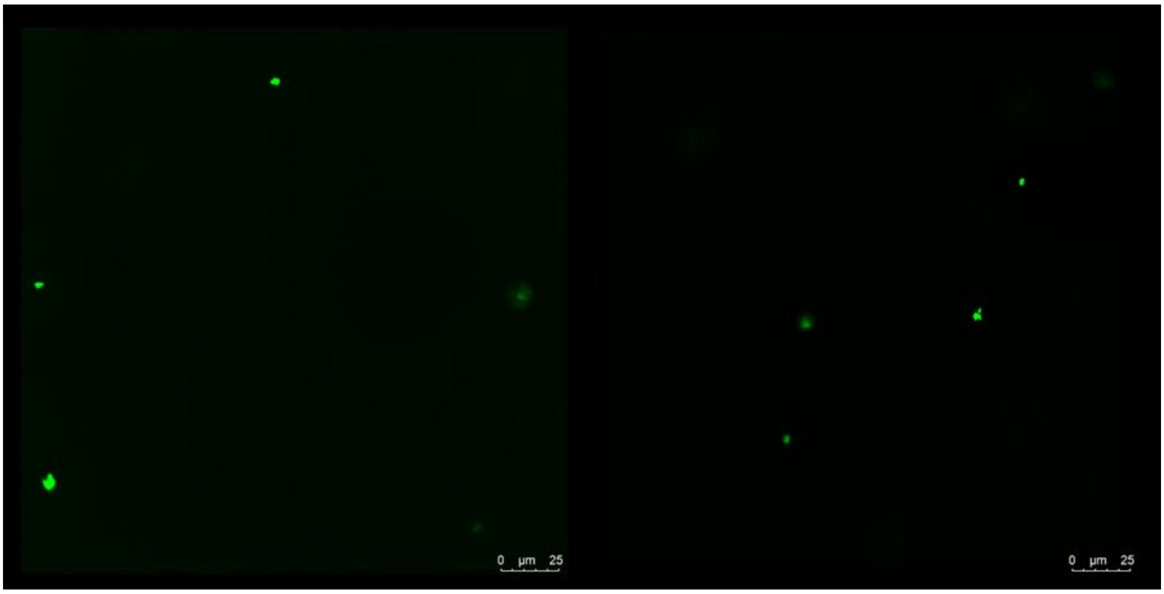

Figure S20 - Confocal images of 0.1 wt% BBS-PMMA samples between 530-620 nm, excited by a 405 nm UV laser. Scale bars: 25 µm.

4.4.4 PET-G

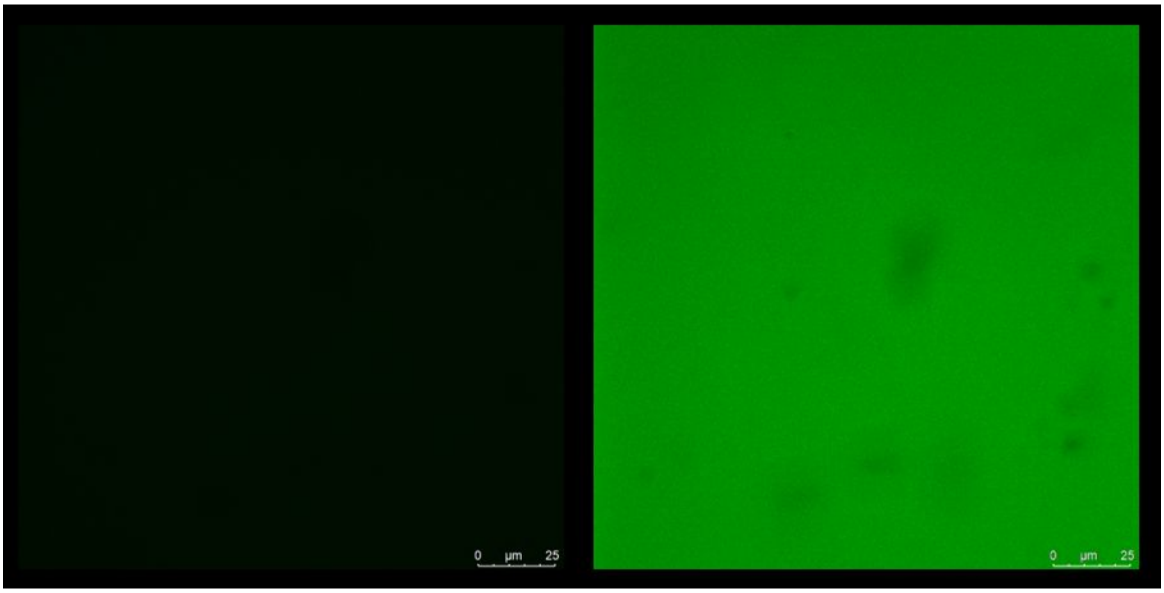

Figure S21 – LHS: Confocal images of 0.1 wt% BBS-PET-G samples between 530-620 nm, excited by a 405 nm UV laser. RHS: Confocal images of 0.1 wt% BBS-PET-G samples between 415-450 nm (monomeric BBS emission range), excited by a 405 nm UV laser . Scale bars: 25 µm.

Confocal images of PET are included in **Section 5.3.2.**

5 Annealing Studies

To investigate the effect of crystallinity of AIEE behaviour, annealing studies were performed in annealable aromatic (PET) and non-aromatic host (PLA). Two hosts were chosen in an attempt to decouple and compare the presence of aromatic groups on the fluorescence response of these systems.

5.1 Annealing Studies on PET-G

To ensure there was little dye migration during heating of a completely amorphous material, a control study was performed by annealing a PET-G samples above 60 ˚C for 60 minutes. No change in

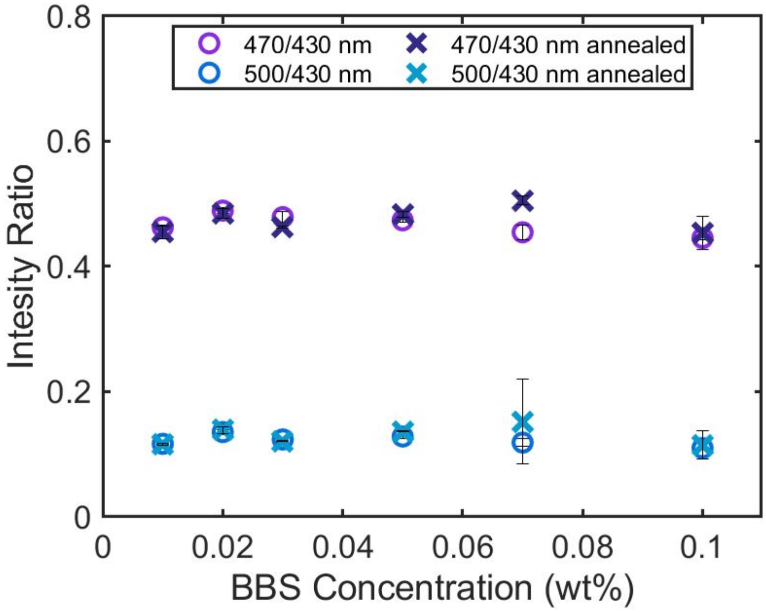

Figure S22 – Fluorescence intensity ratios (**Equation 2**) against increasing BBS concentration for (•) unannealed and (**x**) annealed (60˚C for 60 minutes) BBS-PET-G (dilutions of 0.5 wt%). Fitting produced using the MATLAB curve fitting toolbox. Error bars represent the standard error across 5 samples from the same batch.

fluorescence emission ratios was detected pre- or post- anneal.

5.2 PLA

5.2.1 Crystallinity Studies on PLA

Sample crystallinity was calculated according to **Equation S4** where where ΔH_m and ΔH_c are the melting and cold-crystallisation enthalpies respectively, and ΔH_m° is the melting enthalpy of perfectly crystalline PLA (93 J/g).

$$\%\text{Crystallinity} = \frac{\Delta H_m - \Delta H_c}{\Delta H_m^{\circ}} \#(S4)$$

Table S5 - Crystallinity values for PLA annealed at 80 ˚C from 0 – 60 minutes calculated from DSC (Equation S4).

Time	Crystallinity
0	3.5 ± 0.5
10	3.2 ± 0.1
20	14.5 ± 0.3
40	21.3 ± 0.7
90	27.2 ± 0.4

5.2.2 Fluorescence

Figure S23 - **(A)** Fluorescence intensity ratio (500 : 430 nm) with increasing BBS concentration of BBS-PLA samples (0.1 wt%) at different time points during a 80 ˚C annealing process. Fitting performed using the MATLAB curve fitting toolbox. Error bars represent the standard error from annealing and testing 5 samples per concentration from the same batch. **(B)** Schematic representation of morphological changes during the annealing process, digital photographs of BBS-PLA samples illuminated by 365 nm pre- and post- annealing process.

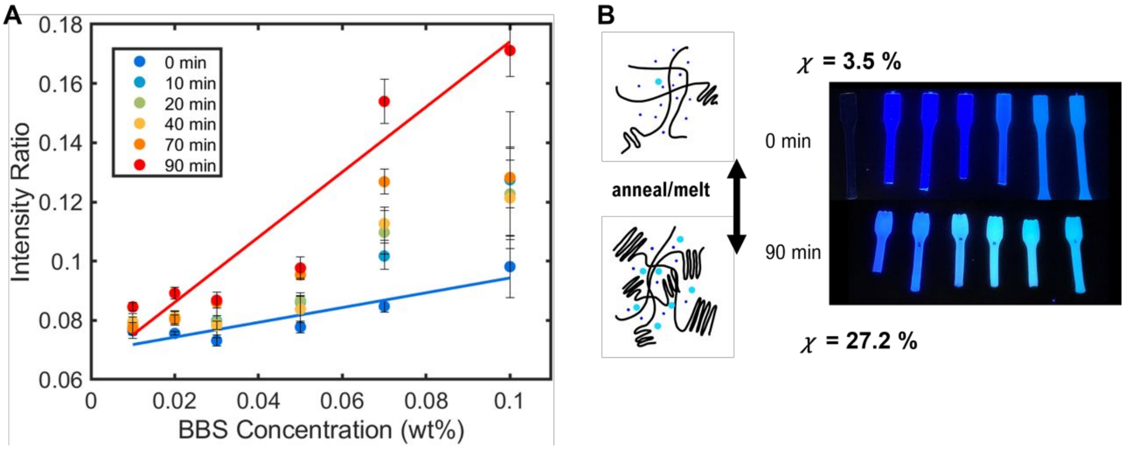

5.2.3 Confocal microscopy

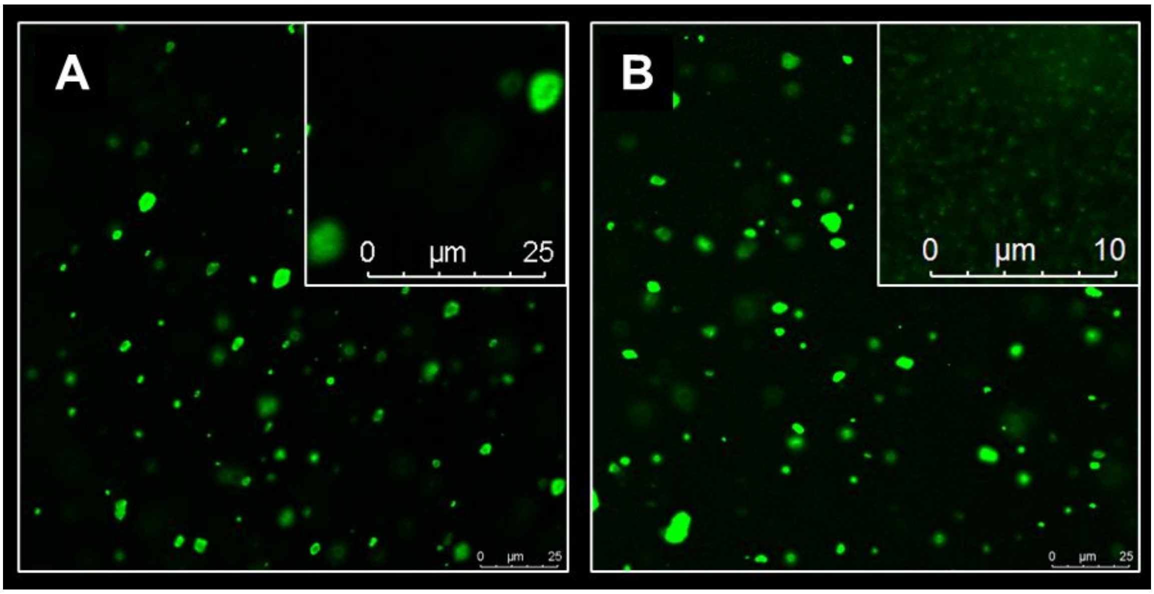

Figure S24 – **(A)** Confocal images of 3.5 % crystallinity (**Table S5**) 0.1 wt% BBS-PLA. Scale bar: 25 µm. Insert: Magnified confocal image of 3.5 % crystallinity 0.1 wt% BBS-PLA. Scale bar: 25 µm. **(B)** Confocal images of 27.2 % crystallinity (**Table S5**) 0.1 wt% BBS-PLA sample. Scale bar: 25 µm. Insert: Magnified confocal image of 27.2 % crystallinity 0.1 wt% BBS-PLA. Scale bar: 10 µm. Images all recorded between 530 – 620 nm and samples excited by a UV 405 nm laser.

5.3 PET

5.3.1 Crystallinity Studies on PET

Sample crystallinity was calculated according to **Equation S4** where where ΔH_m and ΔH_c are the melting and cold-crystallisation enthalpies respectively, and ΔH_m° is the melting enthalpy of perfectly crystalline PET (140 J/g).

Table S6 - Crystallinity values for PET annealed at 100 ˚C from 0 – 120 minutes calculated from DSC (EQ).

Time	Crystallinity
0	3.5 ± 0.5
30	15.4 ± 2.3
60	23.1 ± 3.7
90	25.1 ± 2.8
120	32.0 ± 0.3

5.3.2 Confocal microscopy

The appearance of new aggregates was detected at high anneal times and higher crystallinity through use of confocal microscopy, in agreement with enhanced fluorescence emission re

results.

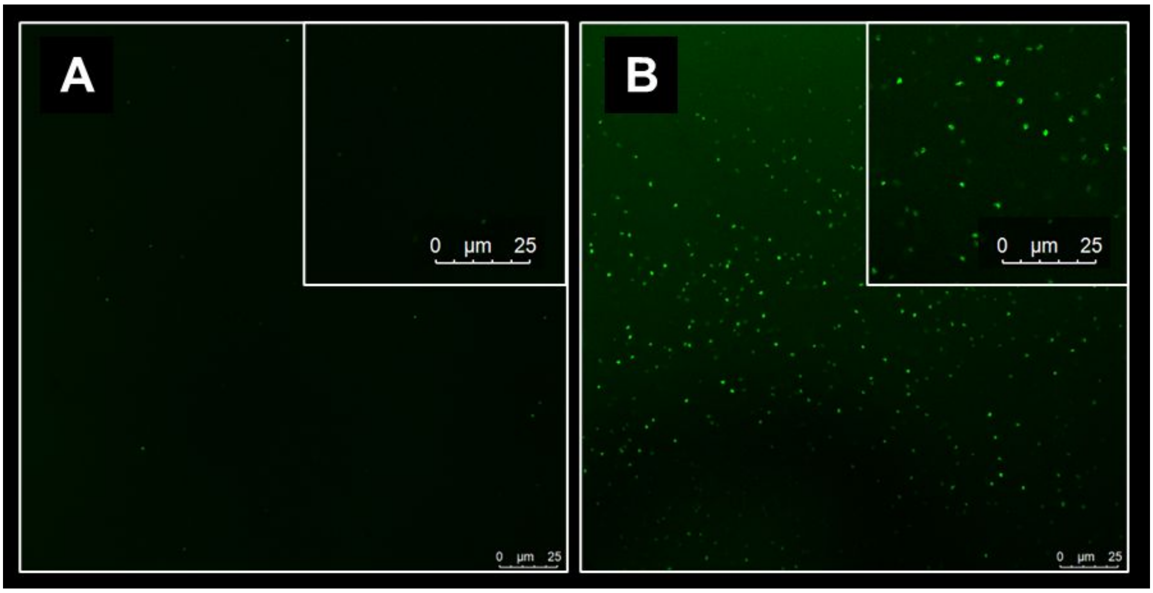

Figure S25 - **(A)** Confocal images of 3.5 % crystallinity (**Table S6**) 0.1 wt% BBS-PET. Scale bar: 25 µm. Insert: Magnified confocal image of 3.5 % crystallinity 0.1 wt% BBS-PET. Scale bar: 25 µm. **(B)** Confocal images of 27.2 % crystallinity (**Table S6**) 0.1 wt% BBS-PET sample. Scale bar: 25 µm. Insert: Magnified confocal image of 27.2 % crystallinity 0.1 wt% BBS-PET. Scale bar: 25 µm. Images all recorded between 530 – 620 nm and samples excited by a UV 405 nm laser.

6 References

(1) Schyns, Z. O. G.; Bennett, T. M.; Shaver, M. P. Recycled Plastic Content Quantified through Aggregation-Induced Emission. **2022**. https://doi.org/10.1021/acssuschemeng.2c03389.

(2) Van Krevelen, D. W.; Nijenhuis, K. Te. Chapter 7 - Cohesive Properties and Solubility. In *Properties of Polymers*; 2009; pp 189–227. https://doi.org/10.1016/B978-0-08-054819-7.00007-8.

(3) Polymerdatabase. *Poly(cyclohexanedimethylene terephthalate)*. https://polymerdatabase.com/polymers/Polycyclohexanedimethylene terephthalate.html (accessed 2022-10-25).

(4) Huang, P.; Pitcher, J.; Mushing, A.; Lourenço, F.; Shaver, M. P. Chemical Recycling of Multi-Materials from Glycol-Modified Poly(Ethylene Terephthalate). *Resour. Conserv. Recycl.* **2023**, *190*, 106854. https://doi.org/10.1016/J.RESCONREC.2022.106854.
